# *Campylobacter jejuni*: targeting host cells, adhesion, invasion, and survival

**DOI:** 10.1007/s00253-023-12456-w

**Published:** 2023-03-21

**Authors:** Leon Kemper, Andreas Hensel

**Affiliations:** Institute of Pharmaceutical Biology and Phytochemistry, University of Münster, Corrensstraße 48, 48149 Münster, Germany

**Keywords:** *Campylobacter*, Adhesion, Antiadhesion, Host–pathogen interaction, Invasion, Virulence factors

## Abstract

**Abstract:**

*Campylobacter jejuni*, causing strong enteritis, is an unusual bacterium with numerous peculiarities. Chemotactically controlled motility in viscous milieu allows targeted navigation to intestinal mucus and colonization. By phase variation, *quorum sensing*, extensive *O*-and *N*-glycosylation and use of the flagellum as type-3-secretion system *C. jejuni* adapts effectively to environmental conditions. *C. jejuni* utilizes proteases to open cell–cell junctions and subsequently transmigrates paracellularly. Fibronectin at the basolateral side of polarized epithelial cells serves as binding site for adhesins CadF and FlpA, leading to intracellular signaling, which again triggers membrane ruffling and reduced host cell migration by focal adhesion. Cell contacts of *C. jejuni* results in its secretion of invasion antigens, which induce membrane ruffling by paxillin-independent pathway. In addition to fibronectin-binding proteins, other adhesins with other target structures and lectins and their corresponding sugar structures are involved in host–pathogen interaction. Invasion into the intestinal epithelial cell depends on host cell structures. Fibronectin, clathrin, and dynein influence cytoskeletal restructuring, endocytosis, and vesicular transport, through different mechanisms. *C. jejuni* can persist over a 72-h period in the cell. *Campylobacter*-containing vacuoles, avoid fusion with lysosomes and enter the perinuclear space via dynein, inducing signaling pathways. Secretion of cytolethal distending toxin directs the cell into programmed cell death, including the pyroptotic release of proinflammatory substances from the destroyed cell compartments. The immune system reacts with an inflammatory cascade by participation of numerous immune cells. The development of autoantibodies, directed not only against lipooligosaccharides, but also against endogenous gangliosides, triggers autoimmune diseases. Lesions of the epithelium result in loss of electrolytes, water, and blood, leading to diarrhea, which flushes out mucus containing *C. jejuni*. Together with the response of the immune system, this limits infection time. Based on the structural interactions between host cell and bacterium, the numerous virulence mechanisms, signaling, and effects that characterize the infection process of *C. jejuni*, a wide variety of targets for attenuation of the pathogen can be characterized. The review summarizes strategies of *C. jejuni* for host–pathogen interaction and should stimulate innovative research towards improved definition of targets for future drug development.

**Key points:**

*• Bacterial adhesion of Campylobacter to host cells and invasion into host cells are strictly coordinated processes, which can serve as targets to prevent infection.*

*• Reaction and signalling of host cell depend on the cell type.*

*• Campylobacter virulence factors can be used as targets for development of antivirulence drug compounds.*

## Introduction

*Campylobacter jejuni* is a Gram-negative, microaerophilic rod-shaped bacterium, which is the most important human pathogenic strain of the 41 species of the *Campylobacter* genus, along with *C. coli* (Parte AC 2022). The World Health Organization (WHO) reports a global incidence of infections in humans ranging from 44 to 93 cases per 10,000 people (e.g., U.K. 93/10,000; Netherlands 58/10,000). It has to be kept in mind that these numbers are estimated cases. At the same time, however, the incidence in less developed countries is known to be much higher than in industrialized countries (WHO 2012; RKI 2018). In contrast to the estimated cases, the number of reported cases in the respective industrialized countries is much lower (e.g., U.K. 9657/10,000 in 2017; Netherlands 3.5/10,000 in 2018, Germany 8/10,000 in 2018) (RKI 2018; Friesema et al. 2022; Public Health England 2018).

In countries among the European Union, approximately 120,000 cases were detected in 2020, which means a decrease of confirmed cases since 2019 of 25.4%, probably because of the COVID-19 pandemic. However, this does not represent a statistically significant decrease in the prevalence of *Campylobacter* enteritis from 2016 to 2020 (EFSA and ECDC 2021). And of course, the influence of the pandemic situation of SARS-CoV-2 has to be kept in mind when analyzing such statistics. Globally, incidences increased steadily in both industrialized and developing countries in the last years (Kaakoush et al. 2015). In this regard, the maximum seasonal peaks of case numbers in Europe are observed around the month of July (European Centre for Disease Prevention and Control 2016). Accordingly, due to the temperature increase of climate change, rising incidences are predicted for the next decades in Northern Europe (Kuhn et al. 2020).

In developed countries, mortality from *Campylobacter* infections is estimated to range between 0.3 and 2.9%, regardless of age (Ternhag et al. 2005). However, in less developed countries, diarrhea is among the clinical manifestations with highest mortality, especially in children. According to WHO, diarrhea is the second leading cause of death in children under 5 years of age worldwide. Annually, 525,000 children die as a result (WHO 2017). In cohort studies of symptomatic children from developing countries, *C. jejuni* was isolated and detected in 8 to 45% of investigated cases, underscoring the relevance of the pathogen in the development of the diarrheal clinical situation (Ruiz-Palacios 2007). However, a record of absolute campylobacteriosis case numbers and resulting mortality rates in developing countries is limited by the lack of diagnostic capacity of laboratories and the lack of surveillance of occurring *Campylobacter* enteritides, and thus is often insufficient (Gahamanyi et al. 2020; Coker et al. 2002).

*C. jejuni* is ubiquitously present in various animal species, partially as a commensal microorganism (RKI 2018). Especially, the handling of raw chicken meat and cross contamination of ready-to-eat food as main transmission source for humans next to inadequately cooked chicken meat are main sources for infections. Typically, Campylobacteriosis peaks during the barbecue season in summer times (Tam et al. 2009; European Centre for Disease Prevention and Control 2016). However, the pathogen can also occur in other livestock, such as pigs, sheep, and cattle (WHO 2012). Consumption of water, contaminated by feces from infected humans, animals, and unpasteurized milk, has been identified as another route of transmission, explaining many local infection outbreaks (Ferrari et al. 2019).

After ingestion by humans, *C. jejuni* enters the intestinal crypts of the lumen, which is lined with viscous mucins (Stahl et al. 2016). This protective layer consists of a hydrated network of highly glycosylated proteins, which protects the underlying epithelium against various aggressive factors (e.g., gastric acid, degrading enzymes, toxins, infiltrating microorganisms) (Herath et al. 2020). *C. jejuni* specifically penetrates and colonizes the mucin layer, eventually establishing contact and invading intestinal epithelial cells (Alemka et al. 2012).

Symptomatic infections with *C. jejuni* are self-limiting after 7 to 10 days. However, they should be accompanied therapeutically by electrolyte and volume substitution. Still, antibiotic treatment is used in many cases (Rosner et al. 2017), which also correlates to the hospitalization rate of about 20% in Europe (EFSA and ECDC 2021). Clinical symptoms are characterized by strong diarrhea, often being bloody and mushy to very watery, abdominal pain, cramps, fever, and fatigue. Early symptoms of these infections include fever, headache, and myalgias. In rare cases, *C. jejuni* infection can trigger autoimmunologic diseases such as Guillain-Barré syndrome (GBS), Miller-Fischer syndrome, or reactive arthritis (Kreling et al. 2020; RKI 2018; Pope et al. 2007; Ajene et al. 2013; Skirrow 1977).

Despite the high and still increasing importance of *Campylobacter* infections and extensive investigations regarding various aspects of *C. jejuni* virulence (e.g., toxin, adhesion to or invasion into the host cell), the exact mechanisms of pathogenesis have not been fully understood yet. However, ongoing research regarding the individual virulence factors has steadily improved the understanding of the development and progression of *Campylobacter* infection in recent years. The most relevant virulence factors are displayed in Table 1.

**Table 1:** The impact of virulence factors discussed in this review on infectivity of* C. jejuni*

Virulence factor	Function	Impact	Source
Motility			
		Loss of flagellar apparatus results in inefficient colonization of mice intestines	(Morooka et al. 1985)
Filament	Propulsion of bacterium	Loss of either FlaA or FlaB results in altered filament construction and loss of motility	(Guerry et al. 1991)
		Loss of flagellins results in loss of function to secret *Campylobacter* invasion antigens as a T3SS	(Konkel et al. 2004)
		Loss of flagellins results in inability to infect chicks	(Nachamkin et al. 1993)
Hook	Connecting filament to basal body	Loss of function to secret *Campylobacter* invasion antigens as a T3SS, if not intact	(Konkel et al. 2004)
Basal Body	Creating rotational force	Loss of function to secret *Campylobacter* invasion antigens as a T3SS, if not intact	(Konkel et al. 2004)
Helical cell body	Basis for reversible winding of the filament around cell body	Loss of helical shape of cell body results in inability to perform darting motility	(Cohen et al. 2020)
Chemotaxis	Directing movement to the epithelial cell/mucus	Loss of chemotactic orientation results in less colonization of mice intestines	(Takata et al. 1992)
		Loss of chemotaxis protein CheY results in non-motile and non-invasive phenotypes	(Yao et al. 1997)
O-Glycosylation of the flagellum	Antigenicity, autoagglutination, biosynthesis of filament, immune evasion, motility	Increased colonization in chicken caecum with higher legionamic acid proportion	(Zebian et al. 2016)
		Loss of or inhibited O-glycosylation results in reduced adherence and invasiveness	(Guerry et al. 2006)
Quorum sensing	Communication within bacterial population	Loss of biosynthesis gene *luxS* for AI-2 results in inhibited transcription of *flaA*	(Jeon et al. 2003)
		Loss of biosynthesis gene luxS for AI-2 results in inhibited motility	(Šimunović et al. 2020)
		Inhibition of AI-2 activity results in reduces biofilm formation and motility	(Li et al. 2021)
Adhesion			
CadF	Adhesion to basolateral fibronectin, initiating invasion	Loss of adhesin results in reduced infectivity of chicks	(Ziprin et al. 1999)
		Loss of adhesin results in reduced adhesion to INT-407 cells	(Monteville et al. 2003; Talukdar et al. 2020)
FlpA	Adhesion to basolateral fibronectin, initiating invasion	Loss of adhesin results in reduced adhesion to INT-407 cells	(Talukdar et al. 2020)
		Loss of adhesin results in reduced adhesion to chicken LMH cells	(Flanagan et al. 2009)
HtrA	Cleaving of tight junctions and adherens junctions as a protease, allowing subvasion paracellular migration	Loss of HtrA results in decreased subvasion efficiency	(Boehm et al. 2012)
	Also acts as chaperone against oxidative stress and misfolded periplasmic proteins	Loss of chaperon function results in decreased adhesion to INT-407 cells	(Bæk et al. 2011)
		Loss of chaperon function results in decreased adhesion to IL-10 deficient mice	(Schmidt et al. 2019a)
N-Glycosylation of over 80 proteins	Influences nutrient household, chemotaxis, stress response, adherence	Loss of N-glycosylation results in reduced adherence, invasiveness in vitro and infectiveness in chicks	(Karlyshev et al. 2004)
		Loss of N-glycosylation results in increased tight junction disruption	(Zamora et al. 2020)
		Loss of N-glycosylation results in reduced adhesion and invasion ability in vitro	(Zamora et al. 2020)
		Loss of N-glycosylation results in reduced colonization of mice intestines	(Szymanski et al. 2002)
Invasion			
*Campylobacter* invasion antigen (Cia)	Induces membrane ruffling of host cell and facilitates invasion	Inhibition of Cia secretion limits severity of *C. jejuni* enteritis in vivo	(Buelow et al. 2011)
	Facilitates survival by modifying *Campylobacter* containing vacuole	Loss of CiaB results in reduced invasiveness into INT-407 cells	(Konkel et al. 1999)
FlaC	Secreted also by T3SS, induces invasion into host cell	Loss of FlaC results in reduced invasiveness into HEp-2 cells	(Song et al. 2004)
Survival			
*Campylobacter* containing vacuole	Protecting the bacterium from lysosomal degradation	Internalization over Fc-receptors and thereby bypassing the natural infection pathway results in lysosomal degradation of the bacteria	(Buelow et al. 2011)
Cytolethal distending toxin (CDT)	Causes cell cycle arrest of the host cell, leading to apoptosis and pyroptosis	Sole application of one of the three parts of the toxin does not show toxicity	(Lara-Tejero and Galán 2000)
		CDT producing strains cause a more severe course of infection in immunodeficient humas	(Smith and Bayles 2006)
		Loss of CdtB results in reduced invasiveness and attenuated immune response in GI tracts of mice	(Smith and Bayles 2006)

This summary of the current knowledge regarding the course of *C. jejuni* infection, and especially the host–pathogen interaction in relation to bacterial virulence factors is intended to connect the previous research in this field to further research for better understanding *Campylobacter*. By understanding the interrelationships of the individual infection phases and virulence factors, new targets for therapy can be identified and their influence on the overall course of disease can be predicted more precisely.

This review thematically follows the course of a human infection with *C. jejuni*: First, we consider how the bacterium reaches the intestinal epithelium by strictly controlled motility. Subsequently, the infection of the host cell by adhesion and invasion is described, leading to the fatal cellular and immunological consequences with tissue destruction, which again results in a final down regulation of the infection.

## Targeting host cells

### Motility of *C. jejuni*: finding its way to the host target

*C. jejuni* is a polar flagellated, helically shaped bacterium. It has high motility and achieves higher velocities in aqueous environments than many other rod-shaped bacteria, such as *Salmonella enteritidis*, *Escherichia coli*, or *Vibrio cholerae* (Ferrero and Lee 1988). The flagellar apparatus of *C. jejuni*, responsible for directed movement and agility, consists of motile filaments attached to a rotating motor via a hook (Glenn-Calvo et al. 1994; Lertsethtakarn et al. 2011).

Several animal studies have examined the potential infectivity of selective mutants in relation to alterations in the motility system of *C. jejuni*: isolates from wild strain FUM158432, which still had intact flagella, but lost chemotactic abilities after treatment with methyl methanesulfonate, colonized laboratory mice worse than the corresponding wild type (Takata et al. 1992). *C. jejuni* mutants that lost filaments or the entire flagellar apparatus due to treatment with mutagens were also unable to effectively colonize the intestinal tract of mice (Morooka et al. 1985). Similarly, chicks could not be infected with *C. jejuni* whose flagellin genes were specifically knocked out completely or partially. Only the entirely motile reference strain was able to colonize the animals (Nachamkin et al. 1993). Also, mutations of a single component of the locomotor mechanism, such as a mutated chemotaxis protein CheY, produced non-motile, non-invasive phenotypes after insertion into the genome of the motile, adherent *C. jejuni* strain 81–176 (Yao et al. 1997). From this, it is concluded that motility of *C. jejuni*, as in other bacteria (Josenhans and Suerbaum 2002), is essential for host colonization and thus virulence (Guerry 2007). In the following, details on the relevant molecular mechanisms for the targeted motility of the pathogen are summarized.

### Motility of *C. jejuni* in the high-viscous environment

Due to the polar flagella and the helical cell body, *C. jejuni* is able to remain motile even in a highly viscous environment and can move easily in such surroundings. Compared to other rod-shaped, flagellated bacteria *C. jejuni* can move much faster in the viscous environment than, for example, *S. enteritidis*, *E. coli*, and V. *cholerae*. Average velocities of 10 to 20 μm/s have been determined for these species at viscosities > 100 cP (for comparison: water 1 cP, olive oil, surface mucus ~ 100 cP, glycerol ~ 1500 cP). In contrast, *C. jejuni* can maintain velocities in the range of 70 μm/s under the same conditions (Ferrero and Lee 1988).

However, no linear decreases or increases in velocity are observed in relation to an increasing viscosity of the medium. Instead, two distinct velocity peaks have been recorded: the first maximum appears similar to that observed in *S. enteritidis*, *E. coli*, and *V. cholerae* and is found at relatively low viscosities of the medium (approximately 1 to 5 cP), whereas a second motility peak is seen at viscosities in the range of 40 cP. This circumstance has been considered to indicate the presence of different, viscosity-dependent locomotion mechanisms (Ferrero and Lee 1988; Shigematsu et al. 1998).

Indeed, *C. jejuni* exhibits different swimming and locomotion patterns within the two motility maxima at the two different environmental conditions as has been shown by video tracking (Shigematsu et al. 1998). The bacterium moves relatively straight in one direction at low viscosity of the medium, as it is typical also for other flagellated bacteria. In this situation, directional changes due to tumbling are rarely observed. The swimming pattern is dominated by flagellar propulsion. At viscosities > 40 cP, *C. jejuni* shows a so-called darting motility, moving rapidly back and forth on straight, short distances in a corkscrew-like manner. In this process, the helical structure of the cell body determines the respective swimming pattern and enables the maintenance of the high motility (Shigematsu et al. 1998; Szymanski et al. 1995; Lertsethtakarn et al. 2011; Cohen et al. 2020).

However, the results of some other studies argue against an essential influence of cell shape on motility: for example, rod-shaped bacteria mutants (e.g., *Helicobacter pylori* mutants) hardly lose motility in high viscosity medium compared to their helical wild types. Nevertheless, cell shape is thought to play an essential role in colonization by these species, albeit here not or only minimally by enhancing motility (Sycuro et al. 2010; Cohen et al. 2020).

Recent studies demonstrate that the two opposing flagella and their respective motors are coordinated during oscillatory motion in such a way that they do not work against each other, but maximum speed can be achieved in viscous environments through cooperativity (Cohen et al. 2020). Again, the helical shape of the bacterium is identified as an important factor, as it enables reversible wrapping of the cell body by the flagellum and thus oscillatory motion in the first place (Cohen et al. 2020). Thus, the leading flagellum unwinds during a change in direction and adopts the function of propulsion, while the opposite-polarity filament wraps around the cell body. In comparison, non-helical *C. jejuni* mutants do not show the back-and-forth movements typical of darting motility, but only forward movements with in-between pauses (Cohen et al. 2020). These pauses result from periods when the leading, wrapping flagellum cannot detach from the mutant's straight cell body. No oscillation of motion occurs (Cohen et al. 2020).

Overall, the motility achieved in high viscosity environment allows *C. jejuni* to effectively penetrate the very viscous mucin layer of the intestinal crypts. *C. jejuni* thus rapidly colonizes the mucus of chickens within the first 24 h after inoculation, becoming poorly excretable and invade thereby host tissues more effectively (Bolton 2015; Smith et al. 2008; Coward et al. 2008). It has to be kept in mind, that the main transmission principle withing chicken flocks is coprophagy (consumption/contact with feces), indicating that persistence of *C. jejuni* in the mucus will not prevent excretion of the pathogen to a high extend (Chaloner et al. 2014). On the other hand, the high viscosity of intestinal mucins slows the movement of *C. jejuni* to the extent that prolonged bacterial-cell contact and consequent increased adhesion increases invasion efficiency (Szymanski et al. 1995). When nested in the mucus, this can also protect the pathogens from damaging exogenous noxae from the intestinal content, e.g., caprylic acid (Hermans et al. 2010).

### The molecular basis of motility: the flagellum

**Fig. 1:**
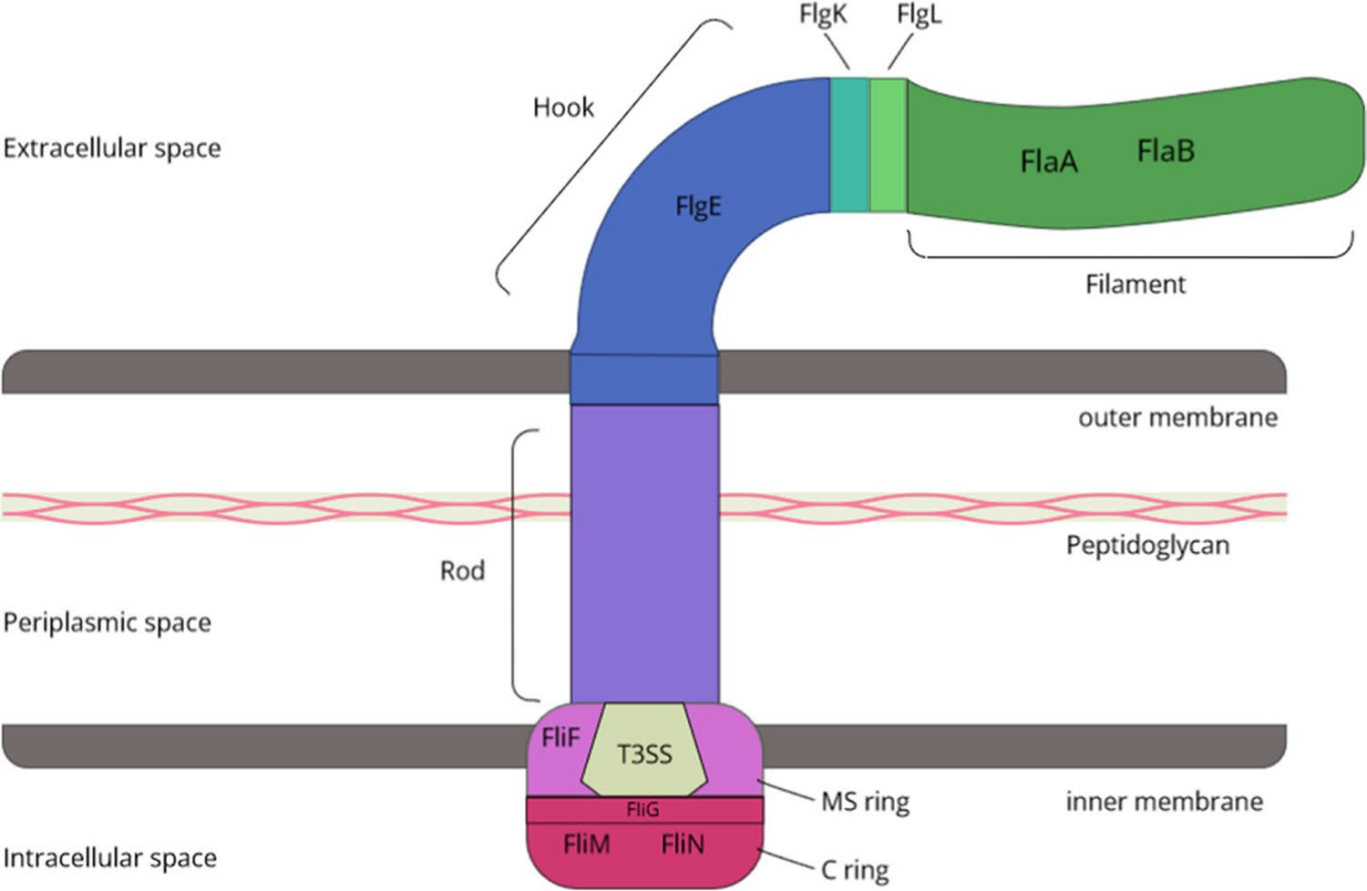
Flagellar apparatus of *C. jejuni.* Internally, the C-ring generates clockwise or counterclockwise rotation through the motor proteins FliM and FliN, which is transferred to the rod via the MS ring. As the connecting piece of the intracellular space with the extracellular space, the rod transfers this rotation to the hook, and consequently to the filament, which generates the locomotion

As in other bacteria, the flagellar apparatus of *C. jejuni* can be divided into three principally distinct units (Fig. 1). The hook-basal body complex protrudes from the intracellular space through the cell membrane and cell wall and is capable of generating rotational force. The hook serves as a flexible joint between the basal body and filament, which acts as a kind of propeller to convert the rotational force generated by the basal body into locomotion (Hong et al. 2018; Fujii et al. 2017). About 30 relatively conserved proteins are found within the complex flagellar structure. Additionally, other factors are involved in the biosynthetic assembly of the flagellum, such as the type 3 secretion system (T3SS), which is responsible for the secretion of flagellum proteins (Lertsethtakarn et al. 2011).

The filament of the flagellum is composed of two highly homologous flagellin proteins, FlaA and FlaB. FlaA represents the major component of the filament, whereas FlaB is only incorporated into the filament in smaller amounts (Guerry et al. 1990, 1991; Nuijten et al. 1990). The different extent of expression of the two flagellins can be explained by transcription through different promoters: The *flaA* gene is transcribed by a σ-28-dependent promoter, whereas the *flaB* gene is transcribed by a σ-54-dependent promoter independently. Using *flaB* or *flaA* mutants, functional flagella consisting of either FlaA or FlaB protein exclusively have been biosynthesized (Guerry et al. 1991; Guerry 2007). The former have the same length of the wild-type filament and slightly reduced motility. The latter are much shorter and produce less motility than wild-type flagella. Thus, expression of both genes is required for maximum motility (Guerry et al. 1991; Guerry 2007).

The filaments are connected to the rotating motor in the cell body of *C. jejuni* by the hook (Glenn-Calvo et al. 1994; Bulieris et al. 2017). This suspension is composed of the polymerized flagellar hook protein, FlgEcj. The FlgEcj amino acid sequence, which is very long compared to FlgE of other bacteria, encodes structures required for stability in the high viscosity environment, preventing flagella from tearing off (Matsunami et al. 2016). Initiation of transcription here, as with *flaB*, occurs through the sigma factor σ-54 (Kinsella et al. 1997; Guerry 2007).

The connection of filament and hook is created by a complex of two ring-like structures, each composed of several units of the proteins FlgL and FlgK. Thus, the hook is attached to the portion of the ring consisting of FlgK, while the counterpart consisting of FlgL integrates the filament into the overall structure (Bulieris et al. 2017; Hong et al. 2018).

On the other side, the hook is connected to a rod originating from the intracellular part of the basal body without any other connecting proteins (Hong et al. 2018). This rod leads to the individual elements of the flagellar base, consisting of the MS ring, the flagellar type III secretion system, the switch complex, and the motor proteins. These in turn are composed of different flagellar protein complexes and each have their own functions (Lertsethtakarn et al. 2011).

The MS ring is a homomultimer of FliF proteins and associated to the inner membrane. Embedded within it is the T3SS, which is primarily responsible for the secretion of *Campylobacter* invasion proteins into the extracellular space. Connected to the cytoplasmic side of the MS ring is the C-ring or switch complex, containing the FliM and FliN proteins, which are important for signal transduction. This serves as the switch of the flagellar motor, which in turn generates the clockwise and counterclockwise rotational force, respectively (Lertsethtakarn et al. 2011; Henderson et al. 2020).

### Chemotaxis for directed motility: navigation to the most favorable milieu

To enter a favorable growth environment, *C. jejuni* uses chemotaxis for navigation. In addition to the flagella, this chemosensory system is an essential component of the directed motility of the bacterium (Bolton 2015). In principle, this involves specific chemical attractants guiding the bacterium in the direction of maximum concentration of the chemoattractant. Negative chemotaxis is also known to cause the bacterium to move in the direction of decreasing concentration of a repellent. As a result, the balance between attraction and repulsion of the chemotactic organism results in localization in the region of the optimal environmental milieu in the respective compartment (Morooka et al. 1985). *Campylobacter* finds this exact optimal environment in the intestinal mucin layer of crypts in mouse and chicken models (Lertsethtakarn et al. 2011; Lee et al. 1986; Beery et al. 1988)), with the mucins themselves in particular being the dominant chemoattractants (Bolton 2015).

### Molecular and mechanistical fundamentals of directional movements

In principle, flagellated bacteria move in a chemotactically determined direction by alternating between the two modes of movement, run and tumbling. A run is characterized by a straight locomotion, while tumbling represents shorter, randomly oriented movements. The counterclockwise or clockwise rotation of the flagella determines the respective mode. A regulated, repeated transition between straight run and re-orientating tumbling eventually results in the directional motility of bacteria (Sidortsov et al. 2017).

Transduction of chemotaxis signals by the corresponding receptors functions in *Campylobacter*, similar to other bacteria, by a two-component system. The two key proteins for this are the membrane-associated histidine kinase CheAY and a cytoplasmic response regulator CheY (Teschler et al. 2017; Zautner et al. 2012; Brás et al. 1999).

*C. jejuni* has chemoreceptors that belong to the group of methyl-accepting chemotaxis proteins (MCPs). They can either be integrated into the membrane and thus detect signals from their environment by the N-terminal periplasmic sensing domain and transmit them to their C-terminal cytoplasmic signaling domain. Alternatively, the receptors are presented freely in the cytosol and react with cytoplasmic proteins. Chemoattractant signals detected in this way are in turn transduced to their C-terminal cytoplasmic signaling domain (Zautner et al. 2012; Li et al. 2014).

**Fig. 2:**
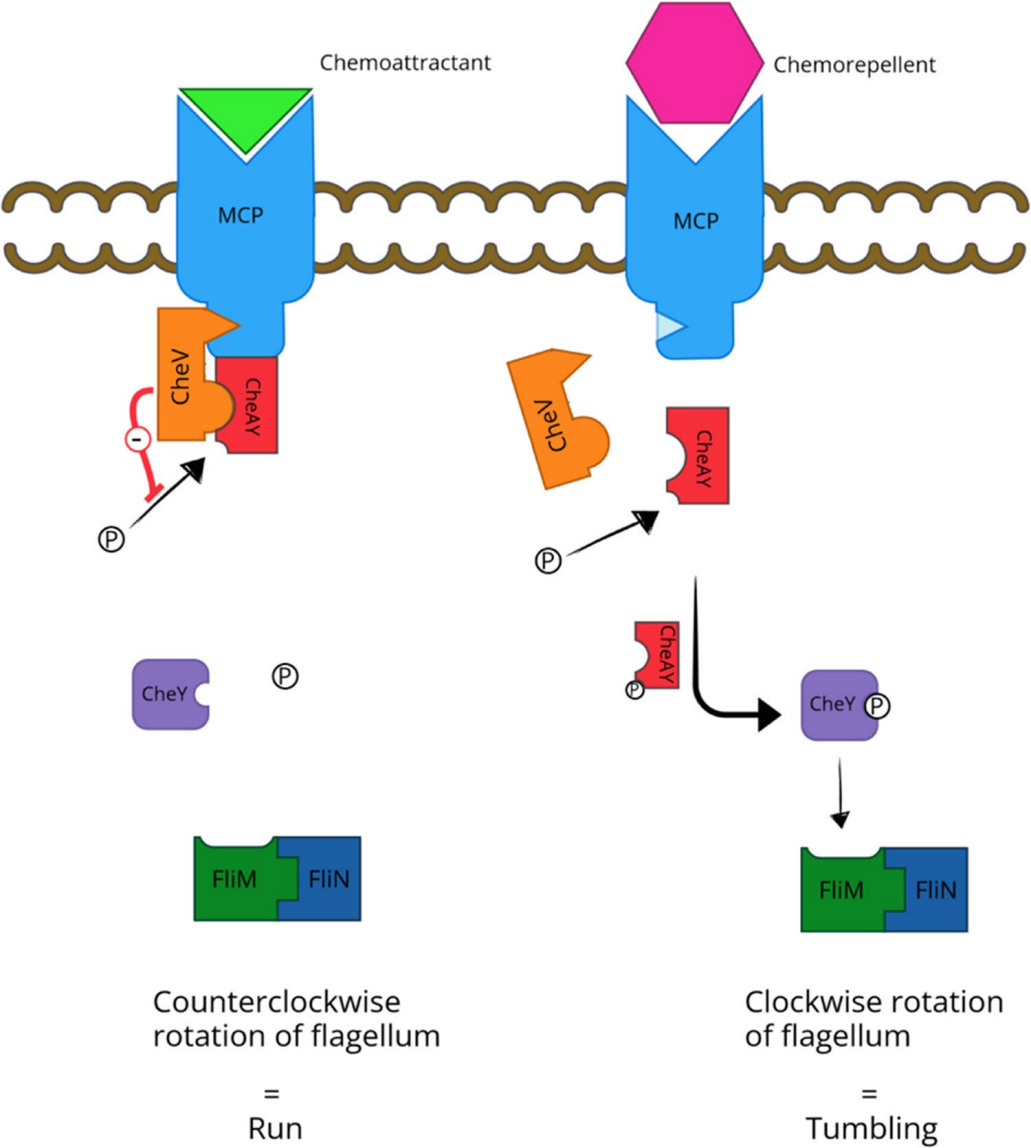
Mechanism of chemotaxis in *C. jejuni*. As soon as a chemoattractant binds to MCP, a trimeric complex of MCP, CheV and CheAY is formed after signal transduction to the C-terminus. This prevents autophosphorylation of the histidine kinase CheAY. Upon contact with a chemorepellent or in the absence of a chemoattractant, CheV no longer associates with MCP. CheAY autophosphorylates and can subsequently transfer phosphate to the response regulator CheY. The latter associates with FliM in the FliM-FliN complex and thus initiates the tumbling

As long as no chemoattractant or a chemorepellent occupies the binding sites of MCPs, no directional movement occurs: The histidine kinase CheAY autophosphorylates to CheAY-P. The phosphoryl group is then transferred to the response regulator CheY (Stewart and VanBruggen 2004). Furthermore, CheAY-P inhibits the dephosphorylation of nascent CheY-P. FliM, a switch protein of the C ring, now binds CheY-P. Only the association of CheY-P with FliM allows a spacial approach of the phosphorylated response regulator to FliN, also part of the C-ring: a FliM-FliN complex forms with bound CheY-P next to this junction (Fig. 2). This is thought to initiate the switching process. The flagellum rotates clockwise; the bacterium is in tumbling mode (Sarkar et al. 2010; Zautner et al. 2012).

In contrast, upon contact of the trimerized MCPs with a chemical attractant and a subsequent activation of the signaling domain at the C-terminus, the latter interacts with a scaffold protein of the bacterium, namely CheV. The histidine kinase CheAY binds to this. The ternary complex thus formed inhibits the autophosphorylation of CheAY. Thus, less CheY is phosphorylated, resulting in less interaction with FliN. Counterclockwise rotation of the flagellum and thereby a run in the direction of the chemoattractant is initiated. Meanwhile, the CheV and CheAY proteins are homologs to CheW and CheA, respectively, which can be found in other bacteria. The former are characterized by a second receiver domain. The biological utility of this extension remains unclear (Zautner et al. 2012; Lertsethtakarn et al. 2011).

However, also other proteins are involved in this mechanism, such as the phosphatases CheZ or FliY, which catalyze the dephosphorylation of CheY, and the methylases CheR and CheB, which can (de)methylate MCPs to adaptively regulate them (Lertsethtakarn et al. 2011).

### Glycosylation of the flagellins influences bacterial motility

A variety of unusual glycans are found on the surface of *C. jejuni* and also a high degree of N- and O-glycosylation of proteins is observed for *C. jejuni*. Glycosylation has a significant influence on the motility and antigenicity of the bacterium, but are also important for bacterial adhesion to and invasion into host cells. The respective fine structures of the oligosaccharide chains are in many cases characterized by the presence of unusual carbohydrates, not commonly found in nature (e.g., altro-, ido-, gulo-, talo-heptoses, *O*-methyl, and *O*-methyl phosphoramidate groups) (Aspinall et al. 1995; McNally et al. 2006).

Flagellin proteins are post-translationally modified by *Campylobacter* spp. through glycosylation (Lertsethtakarn et al. 2011; Logan 2006; Szymanski et al. 2003). Pseudamic acid and its derivatives can be fused to 19 different serine and threonine sites of the flagellins via a structurally diverse *O*-glycosylation system. In this context, the oligosaccharide chains account for about 10% of the mass of the FlaA protein with approximately 6 kDa (Schirm et al. 2005; Szymanski et al. 2003; Thibault et al. 2001).

Glycosylation of flagellins determines, among other things, the antigenicity of these proteins, whereby flagellins themselves represent the immunodominant antigens of the cell surface of *C. jejuni* (Doig et al. 1996; Logan 2006). Furthermore, *O*-glycosylation is essential for the biosynthesis of the functioning filament and thus also significantly affects the motility of the bacterium (Goon et al. 2003; Ewing et al. 2009). This fact, in turn, may have an impact on infection events, as a possible role of glycosylation of FlaA in the context of host colonization has been described: Increased concentration in the caecum of chickens was observed in cj139 mutants that modify flagellins with more legionamic acids than the wild type (Zebian et al. 2016).

Post-translational modification of flagellins further enables *C. jejuni* to auto agglutinate. Corresponding deletion mutants whose flagellins are non-glycosylated or less glycosylated show moderately reduced adherence and invasiveness in experiments on INT-407 host cells (Guerry et al. 2006). In more recent studies, the involvement of glycosylated domains of flagellin subunits in evasion from to Toll-like receptor 5 (TLR5) recognition is observed. The highly conserved epitope recognized by TLR5 consists of an octapeptide (e.g., in *Bacillus subtilis* sequence 86-ILQEVRELVVQ-96), and can be identified in the structure of bacterial flagellins. It is essential for flagella stabilization and therefore also for bacterial motility as a whole (Kreutzberger et al. 2020). *C. jejuni* and other ε-bacteria are able to exchange amino acids in this sequence and can thus evade recognition by the innate immune system. Destabilization after modification of the amino acid sequence is compensated by extensive interactions with outer glycosylated flagellin domains, making modulation possible in the first place without having to lose motility (Kreutzberger et al. 2020).

Over 80 membrane proteins of *C. jejuni* identified to date are modified with bacillosamine-containing heptasaccharides via a highly conserved N-glycosylation system (Szymanski et al. 2003; Cain et al. 2019). The ability to adhere to and invade human epithelial cells is attenuated in *C. jejuni* mutants without these modifications. Also, colonization of chicken intestinal system is also reduced (Karlyshev et al. 2004).

### Phase variation of the flagellum: changing phenotype, motility, and hoax the host

Phase variation results in the reversible exchange of phenotypes resulting from random errors during DNA replication, due to an enormous variability within many sequences (Kreling et al. 2020). Phase variation depends on the presence of intergenic and intragenic hypermutable G/C homopolymeric tracts, leading to changes in flagella, motility, lipooligosaccharides (LOS), and capsule polysaccharides (for a more in-depths review see: Cayrou et al. 2021). Phase variation enables *C. jejuni* to adapt to changes in the environment or to adapt quickly to changes in the respective host system. Thus, phenotypically heterogeneous bacteria can emerge from one clonal bacterial population by adapting to different environmental factors (van der Woude and Bäumler 2004).

Various cell structure genes of *Campylobacter* are also subject to phase variation, including the loci of capsular polysaccharides (CPS) and lipooligosaccharides (LOS), but also those encoding for the structures of the flagellum (Kreling et al. 2020; Guerry et al. 2012).

It has been observed that *C. jejuni* can switch on and off the synthesis of the flagellum by influencing the transcription of the *flaA* gene (Nuijten et al. 1995). The *flaB* gene is not involved in this switch; small amounts of FlaB were also found in non-flagellated phenotypes. Reduction or even loss of flagella may result in advantages for the bacterium under certain circumstances, e.g., in cases were less energy and biosynthetic resources are required or at times when less bacterial motility is needed). In addition, immunoevasive effects, through the loss of flagella as antigenically dominant moieties on the cell surface via phase variation, provide improved survival for the bacterium (Park et al. 2000; Nuijten et al. 1995; Diker et al. 1992). Some gene loci encoding glycosylation of cell structures are also subjected to phase variation: for example, modification of flagella by glycosylation with legionamic acids plays a significant role in the colonization of chickens by *C. jejuni* (Cayrou et al. 2021; Howard et al. 2009).

### Quorum sensing and motility

Quorum sensing (q.s.) is a form of cell–cell communication and describes the ability of individual bacteria to send and receive information within a population. Small-molecule autoinducers secreted by the bacteria function as signaling molecules in this form of communication and, depending on the density of the population, control the expression of target genes: The larger the population of the respective prokaryotes, the higher the concentration of the total secreted autoinducer. Once a threshold is exceeded, this chemical signal is detected by the bacteria themselves, followed by activation or repression of specific genes and signaling (Asfour 2018; Deep et al. 2011).

In *C. jejuni* populations, individuals may communicate by q.s. via autoinducer-2 (AI-2), a furanoylborate diester, for which *luxS* is essentially involved in the biosynthesis. Studies of *luxS*-deficient *C. jejuni* mutants show, among numerous other attenuating effects, a reduction in bacterial motility (Šimunović et al. 2020; Elvers and Park 2002). For example, a 43% reduction in transcription of the flagellin gene *flaA* was observed in *luxS* mutants (Jeon et al. 2003). A significant 4.7% reduction in total motility in *luxS*-deficient mutants has been described in more recent studies, as well as inhibition of motility by modulation of q.s. by essential oils (Šimunović et al. 2020). Capric acid and lauric acid also attenuate *C. jejuni* biofilm formation and motility by inhibiting AI-2 activity, and thus may limit the bacterium's virulence (Li et al. 2021).

However, recent studies indicate that AI-2 in *C. jejuni* represents a metabolic by-product in this species rather than a true *q.s.* molecule, as suggested by the findings above (Ramić et al. 2022). Further studies have to elucidate if AI-2 is a specific autoinducer for *C. jejuni* and whether the motility inhibiting effects observed are a result of interfering with the respective metabolic pathway.

### Concluding remarks on *C. jejuni* motility

For *C. jejuni*, motility is an essential virulence factor. The bacterium reaches the mucus of the intestinal crypts by chemotactically directed movements by means of physiologically influenced locomotion patterns, attracted by mucins and other glycoproteins. Motility is influenced by various mechanisms, such as glycosylation, phase variation and q.s. They allow the bacterium, even in a highly viscous environment, to adapt to the given environmental factors, e.g., by immune evasion or genetic variability, and thus enable effective colonization and adhesion.

## Adhesion: the first step for attacking host cells

### Adhesion and its general role for the infection process

While optimized motility enables improved survivability and host cell targeting, target-specific adhesion to host cells is a further essential step for the initiation of an infection. The specific interaction of *C. jejuni* with host cell structures mostly occurs by adhesins, which are located as outer membrane proteins (OMP) on the bacterial surface. The corresponding molecular interactions may represent protein–protein or protein-carbohydrate interactions. The interaction between bacterial adhesin and eukaryotic target fundamentally complicates the elimination of *C. jejuni* from the host through intestinal propulsion. Moreover, as in certain bacteria and cells, the binding of adhesins to the specific cell receptors can activate signaling pathways that again will modulate the host cell, e.g., in its physiology or regarding possible immune responses. Thus, specific recognition of the host cell and successful adhesion to the same by *C. jejuni* represents a central factor for effective colonization, subsequent invasion, but also persistence of the bacteria (Klemm and Schembri 2000; Ozeri et al. 2001; Rubinchik et al. 2012; Pizarro-Cerdá and Cossart 2006). Conversely, however, targeted inhibition of bacterial adhesion by anti-adhesive agents also offers the possibility of intervening very early in the establishment of the infection (Deipenbrock et al. 2021; Gottesmann et al. 2020a, 2020b; Scharf et al. 2019).

### Adhesion of *C. jejuni*

Adhesion of *C. jejuni* through adhesins is essential for colonization and internalization into host cells. Pili and fimbriae are not involved in mediating these bonds (Bolton 2015; Rubinchik et al. 2012; Konkel et al. 2020).

*C. jejuni* exhibits a wide variety of cell structures that either interact directly with the surface receptors of the intestinal epithelium as “true” adhesins or provide support in the adhesion process as “putative” adhesins (Rubinchik et al. 2012; Konkel et al. 2020). The most studied and confirmed three adhesins of *C. jejuni* are as follows: CadF (*Campylobacter* adhesion to Fibronectin protein), FlpA (fibronectin-like Protein A), which both interact with host cell fibronectin, and JlpA (*Jejuni* lipoprotein A), which interacts on the host cell with heat-shock protein 90-binding (Jin et al. 2001; Monteville et al. 2003; Talukdar et al. 2020).

Insufficient or conflicting studies exist on numerous putative adhesins, such as CapA, major outer membrane protein (MOMP), or PEB3 (Rubinchik et al. 2012).

### Campylobacter adhesin to fibronectin

The CadF protein consists of 319 amino acids and has a mass of 37 kDa. It is encoded by the conserved *cadF* gene, which, like the *flpA*, is ubiquitously found in most *C. jejuni* isolates (~ 95%) (Gharbi et al. 2022; Konkel et al. 1997). It is presented on the surface of the bacterium and binds specifically to fibronectin (Fn) (Konkel et al. 2020, 1997). CadF exists in different forms, due to post-translational processing, leading to changes in the N-terminal domain and conformational changes (Scott et al. 2010).

Studies regarding the importance of CadF for adhesion to the host cell come to different conclusions. On the one hand, recent studies show that CadF is not an essential factor for infection of mice and the absence of CadF in corresponding *Campylobacter* mutants hardly influences the resulting infection outcome. Consequently, other adhesins of the bacterium are thought to have a compensatory function for the lack of CadF. Inactivation of CadF alone is not sufficient to prevent colonization in this case. However, it is enough to mitigate the overall severity of the course (Schmidt et al. 2019b).

On the other hand, binding of *Campylobacter* to Fn via CadF is essential for in vivo infection of chicks: after oral administration of *cadF* mutants, the administered bacteria were not recovered in the fecal contents of the 60 experimental chicks after 7 days (Ziprin et al. 1999). Adhesion of *C. jejuni* with *cadF* knockout to INT-407 cells was similarly reduced by 50 to 90% (Monteville et al. 2003). Recent in vitro studies also confirm the role of CadF and FlpA as major FN-binding proteins that, in their absence, reduce the adhesiveness of *C. jejuni* to INT-407 cells (Talukdar et al. 2020).

### Fibronectin like protein A

The membrane-bound FlpA protein, being the most important fibronectin-binding protein of *C. jejuni* along with CadF, owes its name to its domains, which resemble the fibronectin type III domains of Fn. The respective *flpA* gene, similar to *cadF*, is conserved in various *C. jejuni* isolates (Flanagan et al. 2009; Konkel et al. 2020).The fibronectin type III domains mediate Fn-Fn interactions. Thus, the corresponding domain analogs of FlpA may also interact with host cell Fn in a similar manner (Konkel et al. 2010).

Mutations in *flpA* or disruptions in its expression reduce bacterial adhesion to chicken LMH epithelial cells and to human INT-407 cells. Furthermore, chicken colonization is inhibited by the absence of FlpA (Flanagan et al. 2009; Konkel et al. 2010; Rubinchik et al. 2012). Additionally, recent studies have shown that FlpA and CadF do not functionally complement each other and, that one adhesin cannot fully compensate for the loss of the other. Only in combination the two proteins can confer maximal adherence of the bacterium to the host cell by binding to different sites of the gelatin-binding domain of Fn (Talukdar et al. 2020).

### CadF and FlpA exert variability in expression

The expressed number of Fn-binding proteins is influenced by various factors (Konkel et al. 2020). For example, the expression of CadF and FlpA in *C. jejuni* is downregulated in cases where the bacterium is in an aerobic environment (Guccione et al. 2017). In other studies, upregulation of CadF was observed in case where the temperature was increased from 37 to 42 °C, by exerting oxidative stress, and by adding porcine mucin to the bacteria (Hong et al. 2014; Koolman et al. 2016; Oliveira et al. 2019). The addition of MUC2, one of the most abundantly secreted mucins in the human intestine, in turn inhibits the encoding of CadF, while increasing the expression of the *Campylobacter* invasion antigen B (CiaB), among other proteins (Tu et al. 2008; Corrigan et al. 2017). *C. jejuni* thus responds to changes in the immediate environment of the bacterium by specific modulation of expression of the adhesins, which helps to adapt to the changing environment, continues the infection process and ensures bacterial survival (Tu et al. 2008; Konkel et al. 2020). Increased expression of CadF induced by oxidative stress (H_2_O_2_, 10 mM, 15 min) was not always accompanied by increased adhesion or invasion in these studies. Rather, a secondary function of CadF is thought to be beneficial for the survival in this stressful situation (Koolman et al. 2016).

### *Jejuni* lipoprotein A

The *Jejuni* lipoprotein A (JlpA) protein consists of 372 amino acids with a molecular weight of 42.3 kDa. The lipoprotein is encoded by 1116 base pairs of the *jlpa* gene. JlpA contains two N-glycosylation sites and is thus post-translationally modifiable (Jin et al. 2001, 2003; Kawai et al. 2012). It is a surface-presented, low-immunogenic adhesin that directly interacts with surface heat shock proteins (HSP) 90α and thereby adheres to HSP-90α presenting cells. HSP-90 can be found within the extracellular matrix and on cell surface, is able to modulate cell migration and is formed to a higher extend during cellular stress (Sidera et al. 2004; Clayton et al. 2005; Li et al. 2007). Subsequently, various signaling pathways, including NF-κB, are activated following this interaction (Jin et al. 2003). Inhibitive effects on adhesion to HEp-2 cells have been observed in JlpA deletion mutants or after preincubation with anti-GST–JlpA antibodies (Jin et al. 2001).

### How to get into the tissue: translocation of *C. jejuni*—para- or trans-cellular?

The multifunctional glycoprotein Fn is present in humans either bound, e.g., on basal membranes or resembles a part of the connective tissue matrices of the intestine, or can occur detached in body fluids. There, Fn plays an important role as a mediator in cell proliferation, adhesion and migration (Kolachala et al. 2007).

For interaction with and for invasion into the intestinal epithelium tissue *C. jejuni* migrates into the subcellular space and adheres to basolateral Fn-rich side of polarized intestinal epithelial cells. Entry from the luminal side into the epithelial cells is not preferred by this bacterium (Konkel et al. 2020). The observed invasion efficiency of the bacterium increases significantly once they are given the opportunity to subvade, i.e., invading from the basolateral side of the host cell after para-cellular migration (van Alphen et al. 2008; Monteville and Konkel 2002). Supporting this observation, in microscopic studies, *C. jejuni* was shown to be less effective in invading intact, closed Caco-2 monolayers compared to more spatially accessible Caco-2 cell islands, where subvasion is not hindered by a confluent cell structure (Bouwman et al. 2013). In contrast, older studies describe transcellular translocation of *C. jejuni* to the basal membrane of Caco-2 monolayers by a conclusion based on indirect experimental prove (Brás and Ketley 1999). In case of translocation by the paracellular route opening of the tight junctions (TJ) should have been observed. However, after translocation, no damage to the integrity of the TJ was detected by means of transepithelial electrical resistance (TER) (Brás and Ketley 1999). A significant reduction in TER or cell layer integrity was not measured until 24 h post infection, which is due to the disruption of the cell layer by the *Campylobacter* infection and the subsequent tissue destruction (Brás and Ketley 1999). Transmigrated wild-type *C. jejuni* can be detected under polarized cell monolayers as early as 15 min after infection. However, invasion is often observed 4 to 6 h later, which indicates early transcellular migration. Furthermore, deletion of the *htrA* gene significantly decreases transmigration, while mutants remain highly motile (Boehm et al. 2012).

**Fig. 3:**
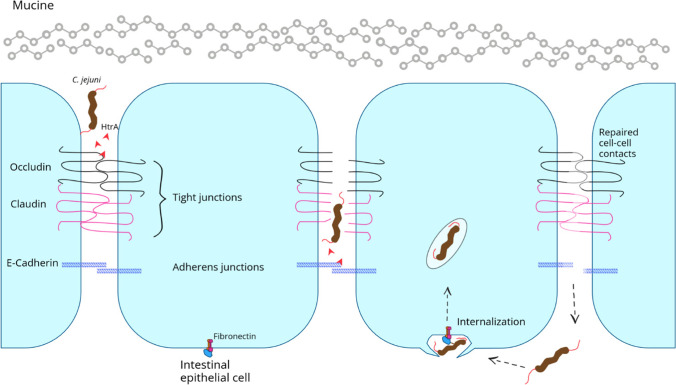
Paracellular transmigration of *C. jejuni* through tight junctions and adherens junctions of intestinal epithelial cells. Secretion of bacterial proteases, such as HtrA, opens cell–cell contacts and the bacterium reaches the basolateral side of the polarized epithelial cells. There, internalization subsequently takes place.

High-temperature requirement protein A (HtrA) is a serine protease expressed and secreted by *C. jejuni* that is capable of cleaving occludin, claudin-8 and E-cadherin. These proteins are important components of the TJ or the usually underlying adherens junctions, which seal the epithelial layer at cell–cell contact sites and allow only selective paracellular transport of small molecules. Secretion of the protease HtrA enables *C. jejuni* to cleave binding proteins of the cell junctions and paves the way for the paracellular route (Boehm et al. 2012; Sharafutdinov et al. 2020, 2022; Zihni et al. 2016; Harrer et al. 2019) (Fig. 3).

A reduction in the translocation ability of *C. jejuni* by mutating HtrA while maintaining motility thus suggests paracellular transmigration, in which the individual cut junction proteins can be rapidly replaced by host cells, thereby not appreciably affecting TER and thus cell-layer integrity (Boehm et al. 2012; Harrer et al. 2019; Sharafutdinov et al. 2020).

The observation of dose-dependent increases in paracellular mannitol flux rates after *C. jejuni* infection in the caecum and jejunum of chicks also supports the disruptive influence of the bacteria on the integrity of the TJ of the intestinal epithelium (Awad et al. 2020). Recent studies confirm this opening of the TJ and, to this end, describe a paracellular parallel transmigration of *E. coli* and *L. lactis* into the basal compartment of Caco-2 cell layers after co-incubation with wild-type *C. jejuni*. After co-incubation with *htrA* mutants, no bacteria of noninvasive strains of *E. coli* and *L. lactis* were recovered on the basolateral side. Microscopically, adhesion of *C. jejuni* and *htrA* mutants to the apical surface of Caco-2 cells has also been recorded, but invasion from this position is still not evident (Sharafutdinov et al. 2022).

*C. jejuni* subvasion is generally dependent on host cell microtubules (MT), whereas actin plays no role in this process (Bouwman et al. 2013).

### The multifunctional HtrA: chaperone and protease activity pave the way to and into cells

In addition to its function as a secreted protease, HtrA exhibits chaperone activity and protects bacteria from oxidative stress by typically degrading aggregates of misfolded periplasmic proteins and inhibiting their formation (Laskowska et al. 1996; Bæk et al. 2011). The function as a chaperone is important for the adhesion ability of *C. jejuni* to its target structures. Thus, in vitro studies indicate a five- to ten-fold decrease in *C. jejuni* adhesion to epithelial cells upon HtrA loss compared to the loss of other adhesins, such as CadF or FlpA. In this regard, a pleiotropic involvement of HtrA in relation to the proper folding of extramembrane proteins is suspected (Bæk et al. 2011).

To assess the specific influence of the chaperone activity on the bacterial adhesion, INT-407 cells were infected with *htrA* deletion mutants. On the other hand, *htrA* S197A mutants, lacking only the protease activity of the protein, were used. While loss of protease function showed a threefold reduction in adhesion compared to wild type, deficiency of the entire protein caused a 20-fold reduction in the bacterial adhesion (Bæk et al. 2011). Similar results were obtained by in vivo experiments in abiotic IL-10-deficient mice: 6 days after infection of the experimental animals with *htrA* S197A, *Campylobacter* loads in the animals were determined to be comparable to those found in animals infected with the wild type. In contrast, loss of HtrA resulted in reduced pathogenicity and a milder disease course (Schmidt et al. 2019a). The chaperone function of HtrA as a factor for successful bacterial adhesion thereby appears to be more essential for the infection event of *C. jejuni* overall than the protease activity of HtrA. Furthermore, it is therefore reasonable to assume that other translocation factors for paracellular migration must exist besides HtrA that are able to compensate for the loss of HtrA (Bæk et al. 2011; Schmidt et al. 2019a).

HtrA, as a virulence factor present in various bacteria, represents a potentially important drug target for fighting Campylobacteriosis due to its dual function as a chaperone and serine protease altogether. It is also conceivable that selective inhibition of the secreted protease HtrA is achievable by substances that cannot penetrate the cell membranes and cell walls of the bacteria. Commensal organisms would thus be able to escape unwanted intracellular HtrA inhibition. However, the more essential aspect of virulence, namely the function as a chaperone, would also be maintained in *C. jejuni* (Skorko-Glonek et al. 2013; Wessler et al. 2017).

### The interaction of CadF and FlpA with Fn: focal adhesion

For successful invasion of many bacteria into their respective host cells, the signaling systems of the eukaryotic cells are activated or manipulated by the pathogens, which in turn triggers re-arrangements of the cytoskeleton and can induce the bacterial uptake (Rosenshine and Finlay 1993). Similarly, specific binding of *Campylobacter* adhesins CadF and FlpA to Fn, which is localized on the basolateral side of the intestinal epithelium, initiates signaling cascades within the host cells, which again promotes the cell invasion and modulates the focal adhesion (FA), among other effects (Konkel et al. 2020). FA represent huge macromolecular assemblies between extracellular matrix and interacting cells. The deletion of FlpA in mutants minimizes the activity of the Rho-GTPase RAC1, which is in part responsible for the so-called membrane ruffling of cells. Membrane ruffling involves the formation of a motile cell surface containing a newly formed matrix, mainly consisting of actin filaments. The formation of such a motile cell surface, because of cytoskeletal restructuring of the cells, is one of the first steps towards cell migration and thus a vulnerable entry point for invasion by *C. jejuni* (Ridley 1994; Eucker and Konkel 2011; Larson et al. 2013). Second, CadF is also associated with the modulation of FA (Klappenbach et al. 2021).

FA, as plasma membrane-associated macromolecular structural complexes, connects the extracellular matrix to the cytoskeleton by integrin binding. They thus transmit, with the participation of a wide variety of proteins, signals to the cell interior for adaptation to environmental factors (Kuo 2014). As a FA adaptor protein, paxillin plays an essential role in the reorganization of the cytoskeleton and subsequent changes in cell shape. Furthermore, paxillin influences other paxillin-binding proteins, such as focal adhesion kinase (FAK), among others, and by doing so, regulates gene expression by activating various MAP kinase cascades (Turner 2000). The MAP kinases are believed to be partly responsible for the induction of inflammatory processes, which again are typical for the clinical symptoms of *Campylobacter* enteritis (MacCallum et al. 2005).

As *C. jejuni* binds to Fn, which again is associated with α5β1-integrin, the bacterium is able to activate signaling cascades within the host cell and thus initiate its own invasion (Konkel et al. 2020; Klappenbach et al. 2021). Thereby *C. jejuni* influences the host cell behavior as a whole by alteration of the structure, composition, and function of FAs. This in turn has consequences for host cell motility, adherence, and wound healing of injured tissues.

Binding of *C. jejuni* increases FA size and in this context cytosolic paxillin is increasingly found at FAs during infection. At the same time the protein density in this area also increases. The exchange of older paxillin proteins for newer, fully functional proteins, namely the paxillin turnover, is simultaneously slowed. The prolonged residence time or reduced turnover of FA proteins thus represents a cause for the enlargement of FAs. It is believed that at a certain size of FA, the resulting host cell motility reaches an optimum. If this optimum is exceeded, as typically occurs during a *C. jejuni* infection, motility decreases in a negatively correlated manner. Consequently, cell migration, e.g., toward injured tissues, is also inhibited (Kim and Wirtz 2013; Klappenbach et al. 2021). Consequently, the inhibition of tissue repair and wound healing, in combination with tissue damage caused by invasion/subvasion, toxin release, and immune responses, will result in massive epithelial barrier defects, electrolyte losses, and severe bloody diarrhea (Boehm et al. 2011).

**Fig. 4:**
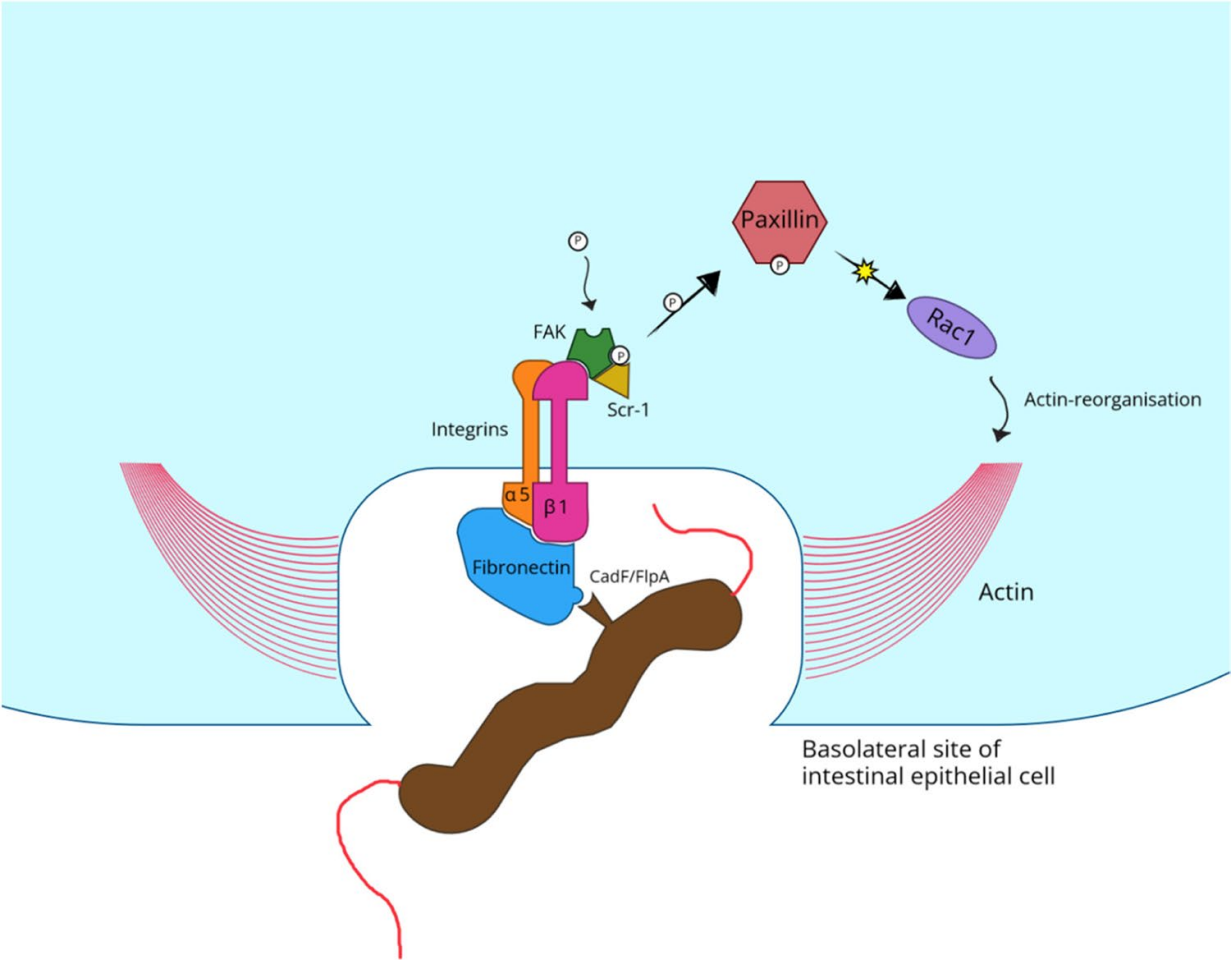
Internalization of *C. jejuni* after adhesion to fibronectin. After subvasion, *C. jejuni* adheres to basolateral Fn of intestinal epithelial cells by interaction with the major adhesins CadF and/or FlpA. Activation of signaling by α5β1-integrins associated to Fn leads to autophosphorylation of the tyrosine kinase FAK. This creates a binding site for the Scr kinase. Scr kinase complex phosphorylates paxillin, which activates Rac1, among others. Finally, Rac1 initiates cellular actin reorganization and membrane ruffling occurs, leading to enclosure of the pathogen

Mechanistically, after binding of integrins to Fn, autophosphorylation of focal adhesion kinase (FAK) occurs (Fig. 4). Thus, a docking site with high affinity for Scr kinase is formed at the phosphorylated tyrosine 397. Subsequently, further phosphorylation at other domains of FAK occurs. The active complex now formed phosphorylates numerous FA proteins and in turn activates them thereby, including paxillin (Mitra et al. 2005; Klappenbach et al. 2021). In addition to FA assembly and disassembly, the active paxillin also influences Rac1 activation by downstream signaling. The interaction of *C. jejuni* with the host cell induces membrane ruffling, which ultimately leads to invasion into the cell (Zaidel-Bar et al. 2007; Klappenbach et al. 2021).

### Glycosylation of *C. jejuni* and bacterial adherence

As mentioned above, posttranslational glycosylation of *C. jejuni* proteins and lipids is of particular relevance in overall virulence. The post-translational modification of the respective proteins by *O*- or *N*-glycosylation contributes a large part to the emergence of different *C. jejuni* phenotypes: the *O*-glycosylation system influences *C. jejuni* motility, immune evasion, and binding to human blood group antigens by modification of flagellins and the MOMP, the modifiable structures identified to date by the system (Mahdavi et al. 2014; Kreutzberger et al. 2020). Among others, *N*-glycosylation modulates the uptake and processing of nutrients, chemotaxis, electron transport, cell stress responses, and, last but not least, various enzyme functions, e.g., nitrate reductase of *C. jejuni* (Cain et al. 2019). However, *N*-glycosylation also plays a special role in the adhesion of *C. jejuni* to host cells (Karlyshev et al. 2004).

Within the highly conserved *N*-linked general protein glycosylation pathway (Pgl), more than 80 proteins have been identified so far in *C. jejuni* which are characterized by a typical heptasaccharide motif (GalNAc-α1,4-GalNAc-α1,4-(Glc-β1,3-)GalNAc-α1,4-GalNAc-α1,4-GalNAc-α1,3-diNAcBac-β1; with diNAcBac as 2,4-diacetamido-2,4,6-trideoxyglucopyranose, Gal as galactose, NAc as N-acetylated carbohydrate, Glc as glucose) attached to asparagine residues (Cain et al. 2019; Duma et al. 2020). The consensus sequence of the glycosylation sites for efficient modifications by bacterial oligosaccharyltransferases is D/E—Y—N—X—S/T, with Y and X being variable amino acids except proline (Kowarik et al. 2006). The biosynthesis and transfer of heptasaccharides to proteins functions with the help of 10 Pgl proteins through several steps, from cytosolic synthesis of diNAcBac, to modification of the protein substrate at the consensus motif by PglB (Cain et al. 2020).

Studies using an in vitro gut-immune co-culture model, which co-cultures enterocytes, goblet cells, and matured dendritic cells in transwell inserts, demonstrates the impact of loss of the Pgl system in *C. jejuni* mutants on the host–pathogen interplay: in addition to increased disruption of TJ, likely due to a concomitant increase in protease expression of the mutants, loss of the bacterial adhesion, and invasion ability could be directly correlated to the absence of *N*-glycosylation (Zamora et al. 2020).

Regarding bacterial adhesion, interactions between *Campylobacter* membrane glycans and superficial host cell structures, such as proteins possessing lectin properties, are suspected. The absence of the *pgl* gene cluster, and thus lack of *N*-glycosylation impairs adherence to host cells (Karlyshev et al. 2004). Similarly, a reduced ability to colonize the intestinal tract of mice by *C. jejuni pglB* or *pglE* mutants has also been observed (Szymanski et al. 2002).

Conversely, galactose-binding lectins are found on the surface of *C. jejuni* that can either bind both α- and β-linked galactose residues of the host cells nonspecifically or interact specifically with one of the two sugar isomers depending on their respective anomeric forms (Day et al. 2013). A lectin that binds fucose and fucosylated structures with broad specificity may also be present on the pathogen surface. Through these lectins, *Campylobacter* can in turn interact and associate with carbohydrates on the host tissue surface (Day et al. 2009, 2013). For example, various *C. jejuni* strains show reduced adherence to Caco-2 cells after co-incubation in the presence of lectins that competitively bind mannose or fucose specifically. However, inhibition of bacterial adhesion by the presence of certain sugars, e.g., mannose, has also been observed (Day et al. 2013, 2009). Thus, it can be assumed that both glycans and as lectins on the surface of *C. jejuni* affect the adhesion to host cells.

### Mannans: potent inhibitors of bacterial adhesion

The above observed finding that lectin-like proteins on the surface of *C. jejuni* contribute to the host–pathogen interaction are further supported by screening of potential antiadhesive glycans*.* In vitro adhesion studies and, to some extent, monitoring of the invasion of *C. jejuni* into Caco-2 cells could be inhibited by co-incubation with mono- or disaccharides, such as D-glucose, D-mannose, and D-maltose. In contrast, the respective L-sugars had no effect on bacterium-host cell interaction (Russell and Blake 1994). Furthermore, reduced adhesion of *C. jejuni* to HEp-2 cells was demonstrated after co-incubation with mannose oligosaccharides. However, the invasion of the respective strains into the host cells was not shown to be affected (Ramirez-Hernandez et al. 2015). Using in vivo experiments by oral application of 0.2 to 0.5% mannose oligosaccharides in diet reduced *Campylobacter* load in the caecum of chickens by 0.5 log levels after 34 days, compared to the control group (Baurhoo et al. 2009). This minor amount of antiadhesive activity in bacterial load are not assessed as clinically relevant. Meanwhile, the number of mucin-producing goblet cells and the length of intestinal villi increased with this dietary supplement (Baurhoo et al. 2009). Feeding chickens with the addition of commercial, specialized feed supplements consisting of mannan-rich fractions of yeast cell walls achieved a maximum reduction in *Campylobacter* concentration of 1.5 log levels after 35 days, according to qPCR analysis (Corrigan et al. 2017) (Corrigan et al. 2017; Rosenquist et al. 2003). Further studies of chicks infected with *C. jejuni* regarding weight loss and weight changes, mortality rates, and blood IL-6 and IFN-γ levels showed no significant differences after supplementation with mannan-oligosaccharides to the non-infected control group, but significant lower ileal and cecal counts of *C. jejuni* (days 24 and 42) than the infected control group (Rostami et al. 2022).

Mechanistically, several ways in which mannan structures may enhance the animal's resistance to pathogenic agents are considered: First, bacterial adhesion to intestinal epithelia is inhibited, possibly via the described lectin desaturation (Day et al. 2013). Second, the protective mucin layer is increased via goblet cell increase. This increase may also allow probiotics adhering to mucin, such as *Lactobacillus*, to find a more favorable growth environment, which in turn may competitively inhibit the spread of pathogenic intestinal bacteria (Baurhoo et al. 2009; van Tassell and Miller 2011; Rahimi et al. 2020). The observed increase in intestinal density of villi also contributes to enhanced host species defenses by providing a preferential colonization site for so-called segmented filamentous bacteria (SFB). SFB are part of the commensal bacterial flora of the chicken. They also inhibit the adhesion of pathogenic agents via competition for nutrients and further by binding to host adhesion receptors that can otherwise be used by pathogens as docking sites to facilitate invasion of cells. Toll-like receptors and ultimately the host immune system are triggered by ligands produced by SFB, which respond with increased production of antimicrobial peptides, recruitment of B and T cells, and secretion of IgA into the lumen, among other responses (Rahimi et al. 2020). Thus, in addition to inhibition of adhesion, morphological changes due to the application of mannan-oligosaccharides and their consequences for the bacterial gut flora of chickens play a role in the decreased colonizability of the intestine.

### The Campylobacter interplay on the adhesion of other bacteria

After co-cultivation of *C. jejuni* with *E. coli* and *L. monocytogenes*, which are also serious food-borne pathogens, *C. jejuni* shows significantly increased adhesion to Caco-2 cells as well as to abiotic surfaces such as polystyrene. This can be caused by the varying composition of the resulting shared biofilms as a basis of adhesion for *C. jejuni*, the availability of metabolic products as food and growth factors, or stress-induced enhancement of adhesion (Klančnik et al. 2020). Certain bacteria included in probiotic supplements, such as various *Lactobacillus* species, competitively inhibit *Campylobacter* adhesion to intestinal epithelial cells through comparatively faster and more efficient interaction with binding sites on host cells (Mohan 2015; Šikić Pogačar et al. 2020). In addition, the effects and influences of SFB elaborated above contribute to reduced virulence of *C. jejuni* in chicken (Rahimi et al. 2020). Besides these factors, it has to be kept in mind that additionally, the immune defense of the host will have a great influence of colonization and virulence, too. It is known that a balanced Th1 and Th2 immune response against *C. jejuni* might explain the bacterial colonization of the caecum and the absence of pathology in infected chickens (Mortada et al. 2021).

Thus, bacterial interactions can increase or decrease the ability of *C. jejuni* to adhere and for virulence. Both approaches thus provide opportunities to attenuate adhesion, either by additionally targeting adhesion-promoting pathogens or by promoting an anti-adhesive bacterial milieu in the intestine.

### Lessons to be learned from *C. jejuni* adhesion strategy

Adhesion of *C. jejuni* to various host cell surface structures, such as Fn, HSP-90α or glycans, by different, independent adhesins represents an initial, but essential step for human infection and enteritis. Binding of adhesins to these cell structures initially ensures specific recognition of and mechanical adherence to the host cell. Subsequent induction of specific intracellular signaling causes changes in FA, cytoskeletal restructuring, and membrane ruffling, which in turn influences the pathogen's invasion. To reach the partially basolateral target structures of polarized cells, *C. jejuni* translocates paracellularly with apparent reversible opening of TJ using HtrA protease. It has to be considered that other translocation factors for paracellular migration must exist besides HtrA that are able to compensate for the loss of this protease. After subvasion, the described binding to the Fn occurs.

Different posttranslational modifications on the part of *C. jejuni* are used to control adhesion, but also bacterial motility. In particular, *N*-glycosylation of outer membrane proteins causes interactions with carbohydrate-binding structures on the host cell membrane. On the other hand, lectin-like structures on the *C. jejuni* surface can in turn bind carbohydrates of the host cell glycocalix. Finally, the bacterial environment of the colonization site can either symbiotically enhance or competitively attenuate adherence.

## Invasion of *C. jejuni*

### The way *C. jejuni* invades into the host cells: zipper or trigger?

After successful subvasion and subsequent adhesion to the relevant host cell proteins, invasion of the cell by *C. jejuni* is initiated. In this process, various mechanisms and invasins are used, which cause uptake or invasion into the intestinal epithelial cells (O Cróinín and Backert 2012). However, under in vitro conditions, it had been shown that only a relatively small proportion of the total *C. jejuni* population gets actually available in the host cells (HT29 cells internalized < 1% of the bacteria in the cell monolayer) (Lobo de Sá et al. 2021).

Mechanistically, bacterial invasion is fundamentally distinguished between “zipper”- and “trigger”-like uptake. The former is defined by pathogen binding to host cell structures with subsequent signaling cascades that induce endocytosis. Membrane rearrangement is limited in zipper-like uptake. In contrast, during the “trigger” mechanism, effector molecules are injected into the host cell, inducing actin-rich membrane ruffles through cytoskeletal restructuring, which internalize extracellular particles by membrane inclusions in a relatively nonspecific manner in the sense of macropinocytosis (Haglund and Welch 2011). The invasion pathway of *C. jejuni* cannot be clearly assigned to these typical entry pathways of intestinal bacteria. There is evidence in the literature and specifically EM images that give some support for both the “zipper” as well as the “trigger” mechanisms of invasion, underlining the concept that *C. jejuni* enters epithelial cells by a unique novel mechanism (O Cróinín and Backert 2012).

The bacterial invasion depends on the host cell proteins of the cytoskeleton.

In many species, bacterial invasion into the host cell depends on the respective cell structures of the host, e.g., actin or microtubulin. However, *C. jejuni* entry into the host cell appears to be partially independent on these proteins of the cytoskeleton: In studies of basolateral invasion of *C. jejuni* into Caco-2 cells, the bacterium was able to invade host cells despite the presence of actin- or microtubulin-inhibiting substances (Bouwman et al. 2013). ATP depletion of the host cells does also not inhibit the invasion of the bacterium. These findings may indicate an overall invasion of *C. jejuni* into host cells that is independent of cytoskeletal restructuring of Caco-2 cells (Bouwman et al. 2013). In contrast, other studies postulate a microtubule-independent secretion of Cia by *C. jejuni*, but at the same time a dependency of the membrane ruffling and thus invasion efficiency on the microtubulin activity of HeLa and INT-407 cells (Konkel et al. 2013; Biswas et al. 2003). It is suspected, based on various observations regarding the invasion mechanism of *C. jejuni* into different host cells, that there is a cell species-specific extent of cytoskeleton involvement in the bacterium's entry process (John et al. 2017).

Fn may accordingly play an important role in the invasion process as a target structure of the *Campylobacter* protein CadF and as a functional interface for cell structure reorganization (Klappenbach et al. 2021).

Inhibition of dynein, a protein responsible for transport of vesicles by microtubules from the cell surface to the nuclear environment, reduces *C. jejuni* uptake. Furthermore, co-localization of the pathogen with dynein has been observed microscopically (Hu and Kopecko 1999). Like dynein, also the endocytosis protein clathrin is involved in *C. jejuni* invasion in human and avian cells. This has been shown by use of the clathrin inhibitor chlorpromazine, which leads to significantly reduced *C. jejuni* invasion into HT29 and 8E11 cells (John et al. 2017).

Similarly, *C. jejuni* invasion was initially thought to depend on caveolae, which are a particular form of cholesterol- and glycosphingolipid-rich plasma membrane sections called lipid rafts (Watson and Galán 2008; Konkel et al. 2013; John et al. 2017). However, studies focusing on these results showed that depletion of caveolin-1, a major component of caveolae, does not prevent *C. jejuni* internalization. Rather, it plays a role in signaling induced by the interaction of *C. jejuni* at host Fn (Konkel et al. 2013; John et al. 2017).

### Secretion systems of *C. jejuni*

As the direct association between motility of *C. jejuni* and its invasion is obvious, it has been discussed that the flagellum might also be used as a secretory device for invasion-associated effector molecules (O Cróinín and Backert 2012). In fact, the flagellum represents a type iii secretion systems (T3SS) by definition and this flagellum-associated protein secretion system represents a transportation system for mediating invasion into the host cell (O Cróinín and Backert 2012; Puhar and Sansonetti 2014; Neal-McKinney et al. 2010; Desvaux et al. 2006). T3SS are characterized by using an ATPase-dependent process in which proteins after host cell contact are centrally shuttled through a hollow filament across the cell envelope into the extracellular space (Desvaux et al. 2006). Invasins, in our case called *Campylobacter* invasion antigens (CiaB, CiaC) are secreted via T3SS, which are required for optimal invasion into epithelial cells (Konkel et al. 2004; Christensen et al. 2009). While mutations of various proteins involved in the correct filament assembly result in the absence of the secretion of Cia, the presence of the flagellins, FlaA or FlaB, is sufficient for functional secretion of the invasins (Konkel et al. 2004). Similarly, an intact basal body and the hook are required for secretion (Konkel et al. 2004).

In addition to T3SS, also a type VI secretion system (T6SS) is found in different *C. jejuni* strains with increasing, regionally varying prevalence. Built like the inverted tail of a bacteriophage, the T6SS is embedded in the cell membranes of various Gram-negative bacteria and is thus capable of delivering effector molecules to other prokaryotes or eukaryotes by penetration of cell membranes (Coulthurst 2019; Liaw et al. 2019). This may play a role in interbacterial competition, but could also contribute to adhesion to the host cell, subsequent invasion, and in vivo colonization (Lertpiriyapong et al. 2012; Coulthurst 2019). However, no effectors have yet been identified that are not in themselves part of the secretion system, such as TssD, which forms the needle-like structure of the T6SS (Liaw et al. 2019). Bioinformatic analyses of the *C. jejuni* 488 strain found potential effectors downstream of the T6SS gene locus in the newly defined *Campylobacter jejuni* pathogenicity island-1 (CJPI-1) there. In addition to putative nucleases and NAD^+^ glucohydrolases, presumed anti-eukaryotic and anti-prokaryotic effector sequences have been identified, which could thereby enhance fitness in the context of microbiological competition in the intestinal space (Robinson et al. 2021).

### Campylobacter invasion antigens

In 1999, a *Campylobacter* invasion antigen, namely CiaB, was identified for the first time and associated with the T3SS as a secreted effector due to the similarity to already known type III secreted proteins of other bacteria. In vivo studies indicate that secretion of the Cia proteins contributes to the severity of *C. jejuni-*mediated enteritis (Buelow et al. 2011). Furthermore, in the absence of *ciaB* in mutants, no reduced adhesion to the host cell was detected, but an inhibition of the invasion efficiency of the bacterium into the host cell was observed (Konkel et al. 1999). Cia proteins are expressed upon contact of *C. jejuni* with intestinal cells and can be induced by supplementation of cultivation media with bovine calf serum or serum supplements obtained from other species (Rivera-Amill and Konkel 1999). The T3SS itself is considered to be a contact-dependent secretion system (2001; Scherer and Miller). Upon host-cell contact of *Salmonella typhimurium*, the effectors of the T3SS, initially localized in the bacterial cytosol, were observed to be secreted 10 to 90 s after docking to the target structures (Schlumberger et al. 2005). A similar pathogen-host contact-dependent secretion is also suspected for the T3SS of *C. jejuni*. Effector delivery after completed internalization has been ruled out (Neal-McKinney and Konkel 2012). Due to the contact dependency of the secretion system and without own adhesion potential of the effectors, host-proximal secretion of invasion-mediating antigens is directly dependent on the spatial association between *C. jejuni* and the intestinal epithelium. Thus, the presence of the major adhesins, like FlpA, is essential for the release of effector molecules by the T3SS (Talukdar et al. 2020; Konkel et al. 2020).

Effector molecules secreted into the host cell cytosol by the T3SS have previously been reported to include CiaB, CiaC, CiaD, CiaI, FlaC, and FspA (Neal-McKinney and Konkel 2012; Samuelson et al. 2013). Studies have shown that the delivery of specific effectors, such as Cia, is dependent on the particular host cell type with which the bacterium gets into contact (Neal-McKinney and Konkel 2012).

CiaD, after secretion into the host cytosol, binds the intracellular protein IQGAP1, which subsequently interacts with Rac1 to mediate the actin restructuring of the cytoskeleton. CiaD also keeps the Rho GTPase active by excluding RacGAP1, a Rac1 regulator, from the IQGAP1-binding complex, thereby reducing Rac1 inhibition (Negretti et al. 2021).

Through an IQGAP1-independent pathway, CiaD induces activation of extracellular-signal regulated kinases (ERK) 1/2, which in turn can phosphorylate and thus activate cortactin. Subsequently, as after interaction of CiaC with the host cell, actin cytoskeleton restructuring and membrane ruffling occurs, allowing *C. jejuni* to enter the host cell (Talukdar et al. 2020; Neal-McKinney and Konkel 2012; Negretti et al. 2021).

Altogether, both adhesins (e.g., CadF and FlpA) and invasins are required for optimal membrane ruffling and thus maximum invasion efficiency. In addition to modulating focal adhesion, adhesins also create the spatial proximity required for the effective invasin interaction with cell structures (Klappenbach et al. 2021).

CiaI, on the other hand, is associated with intracellular survival of *C. jejuni* in the host cell. Internalization of *C. jejuni* into the cell results in the formation of a so-called *Campylobacter* Containing Vacuole (CCV), which may be modified by CiaI. Mechanistically, effects of exclusion of vacuole-aging proteins, retention of certain markers that would cause vacuole aging, or acquisition of certain proteins that may prevent aging are discussed (Buelow et al. 2011).

Independent of pathogen-host contact and associated cell signaling, FlaC and FspA proteins are secreted by the flagellar apparatus. FlaC, despite homologies to the flagellins FlaA and FlaB, has no bearing on *C. jejuni* motility, but reduces invasion frequency in deletion mutants (Baqar et al. 2008; Song et al. 2004). The functions in the infection process of FspA1 and FspA2 are largely unknown. Recombinant FspA2 induces apoptosis when bound to intestinal epithelial cells in vitro, but this effect was not observed with FspA1 (Poly et al. 2007).

### Autophagosome: a potential route of the bacterium into the cell?

In many cases, bacterial entry into the host cell triggers the host defense system for elimination of intracellular pathogens (Baxt et al. 2013). Especially autophagy can lead to effective degradation of bacterial cells by insertion into the autophagosome system and subsequent degradation by lysosomal fusion (Deretic 2011). Interestingly, for *C. jejuni*, it was found that the bacterium did not colocalize with autolysosomes or autophagosomes, indicating that this system does not contribute too much to the bacterial clearance. In contrast, the autophagy system of *C. jejuni* seems to be involved in the invasion system of the bacterium, which again facilitates the infection (Fukushima et al. 2022). An increase in the concentration of the cellular autophagosome protein LC3-II, even before internalization of the pathogen occurred, was detected. This could mean that *C. jejuni* is able to stimulate this autophagocytosis factor from outside the cell to influence the entry process in favor of invasion. After binding of *C. jejuni* to host Fn via CadF and the following activation of Rac1, enhanced recruitment of the cytosolic autophagosome protein LC3 to the entry site with subsequent successful invasion is described (Fukushima et al. 2022). These processes led to the suggestion that the autophagocytosis signaling pathway represents a potential entry point for *C. jejuni* into the host cell (Fukushima et al. 2022). Altogether, it is suggested that targeting the autophagy system might be a potential target for inhibiting the invasion of major foodborne-causing pathogens like *C. jejuni* (Fukushima et al. 2022).

## *C. jejuni* in the host cells: how to survive

### General aspects on the intracellular survival

After successful invasion into the intestinal host cells, *C. jejuni* is able to survive within the intracellular space, depending on the host cell type. The bacterium can be metabolically active for 24 to 72 h, but can also seek his way to penetrate into deeper tissues (Tegtmeyer et al. 2021; Watson and Galán 2008; Campana and Baffone 2020). In this intracellular phase, *C. jejuni* influences the intracellular regulatory processes of the host cells by different factors (e.g., CDT, Cia). At the same time, the pathogen itself is also influenced by the altered environmental factors and adapts in a reactive way (Watson and Galán 2008; Hickey et al. 2000; Buelow et al. 2011). Furthermore, the pathogen persists even after phagocytosis by monocytes, can induce inflammatory processes, and can eventually induce host cell apoptosis (Hickey et al. 2005). The apoptotic and inflammatory processes, together with disruption of cell–cell contacts and inhibition of wound healing, result in intestinal barrier dysfunction. Electrolytes and water leak out, which again cause bloody diarrhea in the patient (Butkevych et al. 2020). Inhibition of wound healing by *C. jejuni* and interaction with FA could further exacerbate this problem (Klappenbach et al. 2021). This damage may in turn allow superinfection by nonpathogenic pathogens to opportunistically invade the injured structures and exacerbate or, in rarer cases, chronify the inflammation (Campana and Baffone 2020).

Overall, the intracellular career of *C. jejuni* appears less extensively studied compared to the previous elaborated virulence factors of motility, adhesion, and invasion. In particular, the pathogenesis is difficult to elucidate without the appropriate in vivo models (Young et al. 2007). However, in recent years, some progress has been made regarding various mouse models designed to emulate human pathogenesis (Heimesaat and Bereswill 2015). For example, in newly described models, the mouse immune system is sensitized to LOS to resemble more closely a corresponding immune response of a human. Normally, this response is significantly reduced in experimental animals due to a weaker TLR-4 response (Mousavi et al. 2020). Nonetheless, as *C. jejuni* exhibits some exceptional features in its virulence, particularly in human infection, it has yet to be shown that the respective animal models developed can represent the actual course of infection in humans in a whole.

### Campylobacter containing vacuole and its influence on survival

When *C. jejuni* enters the host cell, the particular invasion pathway may influence the intracellular survival of the bacterium, as for example autophagolytic degradation of the pathogen can be avoided. In human infections, *C. jejuni* — after entering the cell — remains in a membrane-enclosed compartment, the so called CCV (Watson and Galán 2008). The CCV is distinct from lysosomes and does not co-localize with lysosomal protein markers, such as cathepsin B, indicating a distinct maturation process from the classical endocytic pathway (Buelow et al. 2011). On the other side it has been reported that immediately following *C. jejuni* cell uptake, the CCV displays markers for early endosomes, such as early endosomal antigen-1 (EEA-1) (Buelow et al. 2011).

In addition, a tight microtubule- and dynein-dependent association of the CCV with the host cell Golgi apparatus has been observed without acquisition of Golgi markers (Watson and Galán 2008). Fusion of the CCV with the lysosomes does not occur, which means that the pathogen thus bypasses the host cell defense system. This is partly due to the secretion of CiaI by T3SS, which prevents the delivery of CCV to lysosomes (Buelow et al. 2011). Experimental internalization of *C. jejuni* by Fc receptors (receptor type binding to the Fc part of immunoglobulins) led to the degradation of intracellular *C. jejuni* via lysosomes, which indicates that different invasion pathways lead to different degradation and defense pathways against *C. jejuni.* (Watson and Galán 2008; Buelow et al. 2011). Within the CCV, *C. jejuni* is transported, through interaction with dynein, along microtubules into the perinuclear environment (Hu and Kopecko 1999). The further course of the pathogen after translocation is still largely unexplored (Young et al. 2007).

### Cytolethal distending toxin and survival: an important element for the pathogenesis of campylobacteriosis

Once intracellular *C. jejuni* will secret the genotoxin cytolethal distending toxin (CDT**)**, leading to cell cycle arrest, cell swelling and cell distension (Lara-Tejero and Galán 2000). The tripartite protein toxin CDT, a member of the DNase I protein family, is encoded by three genes, *cdtA*, *cdtB*, and *cdtC*. It promotes cytotoxicity of the pathogen and can be assessed as a strong virulence factor of *C. jejuni*. CDT can cause DNA damage after translocation to the nucleus, which in turn results in the induction of DNA damage response and after cell cycle arrest leads to apoptosis. The nuclease activity of CdtB found in vitro is likely responsible for DNA damage and subsequent G2/M-phase arrest (Lara-Tejero and Galán 2000). However, DNase I function has not yet been fully confirmed, in contrast to the DNA damage that takes place (Méndez-Olvera et al. 2016; Pons et al. 2019). The two other subunits of CDT, CdtA and CdtC, are regulators for the interaction via lipid rafts with the cell membrane. In cases where purified CdtA, CdtB, or CdtC were applied, no cell toxicity was observed (Lara-Tejero and Galán 2000). In contrast when combined, CdtA, CdtB, and CdtC interact with one another to form an active tripartite holotoxin that exhibits full cellular toxicity (Lara-Tejero and Galán 2000). Therefore, it is proposed that CDT is a tripartite toxin composed of CdtB as the enzymatically active subunit and of CdtA and CdtC as the heterodimeric B subunit, required for the delivery of CdtB (Lara-Tejero and Galán 2000) Extracellular CDT can be internalized by binding to a surface receptor, which requires intact lipid rafts, where CdtA and CdtC can interact with the cell membrane and enable the translocation of the holotoxin across the cell membrane (Méndez-Olvera et al. 2016).

CDT is transported towards the nucleus with the involvement of the cytoskeleton. This is indicated by the reduced effect of CDT after inhibition of the microtubule dynamic and actin formation (Méndez-Olvera et al. 2016). In addition, however, other apoptotic mechanisms have been observed that are not dependent on the secretion of CDT (Hickey et al. 2005; Pons et al. 2019).

In addition to or instead of apoptosis, CDT can induce pyroptosis in the host cell, which is another form of programmed cell death (Gu et al. 2022). Ion fluxes through plasma membrane pores lead to loss of cellular ion gradients, increase in osmotic pressure, and eventually osmotically induced lysis. The formation of the pores is dependent on the proteolytic protein caspase-I and the resulting rapid disruption of the plasma membrane, in contrast to the process of apoptosis, releases intracellular proinflammatory mediators, which in turn can activate additional immune cells in the environment (Bergsbaken et al. 2009). Whether the CDT-induced pyroptosis has been mistaken in the past for apoptotic processes or whether both mechanisms of programmed cell death occur simultaneously needs to be assessed by further studies (Gu et al. 2022; Bergsbaken et al. 2009).

The CDT toxin appears to be an important element for the pathogenesis of campylobacteriosis, as *C. jejuni* strains producing CDT elicit a more severe course of infection in immunodeficient humans (Smith and Bayles 2006). CdtB null mutants showed reduced invasiveness and attenuated immune response in the GI tract of mice (Smith and Bayles 2006). Toxin action and subsequent epithelial cell death eventually leads to destruction of intestinal wall integrity, which then results in symptomatic diarrhea (Smith and Bayles 2006). It has to be mentioned that CDT (also *C. jejuni* secreting strains as Cj81-176) can promote colorectal cancer (He et al. 2019).

### *C. jejuni* not only inside the intestinal cell, but also in monocytes: bacteremia in humans?

The survivability of *C. jejuni* within monocytes was postulated early on: According to this, the pathogens are able to remain viable intracellularly for at least 6 days (Kiehlbauch et al. 1985). This circumstance may increase the risk of bacteremia in humans (Wassenaar et al. 1997). However, later studies limited these results in a way that a large proportion of the activated monocytes were very capable of efficiently killing *C. jejuni*. Only 10% of the monocytes considered from donated human blood were unable to eliminate the pathogen after successful uptake (Wassenaar et al. 1997). Later, intracellular survival of the pathogens over a 7-day period within human 28SC monocytes was confirmed by further viability assays. Compared with non-phagocytozed *C. jejuni*, the phagocytozed pathogens live even up to four days longer, in agreement with the original study. Moreover, replication of these bacteria within monocytes predominates until 48 h post infection before subsequently decreasing in number (Hickey et al. 2005; Kiehlbauch et al. 1985).

Indeed, DNA, corresponding to certain *Campylobacter* proteins have been recovered in myelomonocytes circulating in humans. PCR and Southern blot analyses revealed that 30% of healthy study participants and 50% of GBS patients studied had *C. jejuni* DNA in their monocytes. Surviving pathogens could not be isolated from the monocytes (van Rhijn et al. 2002).

Involvement of monocytes in the development of GBS has only been demonstrated by the increased phagocyte numbers or increased monocyte-leukocyte ratios in diseased patients (Huang et al. 2018; Li et al. 2018). Thus, it could be inferred that monocytes, which take up *C. jejuni* but are unable to kill them efficiently, may transport the still-active pathogens or their antigenic components as vehicles into the circulating bloodstream. Systemic infections and inflammatory responses can be the consequences of this process.

### Adhesion, invasion, and intracellular phase: the inflammation cascade as the subsequent process

Because of the strong cell destruction after *Campylobacter* infection, the release of inflammatory chemokines occurs. In particular, the upregulation of transcription of NF-κB-dependent chemokines, such as CXCL1 (chemokine[C-X-C]motif ligand 1), CCL3/CCL4 (CC-chemokine ligand 3 resp. 4), CCL2 (CC-chemokine ligand 2), and CXCL10 is observed. Chemotactically, these mediators in turn attract immune cells to the infected tissue (Hu and Hickey 2005).

Activation of cellular NF-κB signaling pathways, as by other partially harmless bacteria, may occur by interaction of pathogen-associated molecular patterns (PAMPS) with cellular Toll-like receptors, and thus represent a more general tissue immune response to infection. Induction of NF-κB and subsequent immune responses by an alpha-kinase-1-dependent (ALPK1) signaling pathway is described as more specific to *Campylobacter* enteritis. *C. jejuni* secretes ADP-heptoses and similar heptose phosphates that activate ALPK1 upon infection, independent of type III and type IV secretion systems (Cui et al. 2021). A similar inflammatory mechanism via ALPK1 has also been observed during *H. pylori* infections (Cui et al. 2021; Bauer et al. 2020).

Host immune cells involved in the resulting inflammation mainly include neutrophil granulocytes, monocytes, macrophages, dendritic cells, natural killer cells, T cells, and B cells (Callahan et al. 2021).

Migrated neutrophil granulocytes counteract *Campylobacter* infection early by means of phagocytosis, release of antimicrobial proteins as well as proinflammatory substances, and laying out neutrophil extracellular traps (NET). Cytotoxic NET-like structures were also recovered in crypt abscesses of infected experimental animals, indicating the involvement of neutrophil granulocytes in intestinal damage (Callahan et al. 2021; Brinkmann et al. 2004; Li et al. 2020). *C. jejuni* also affects the differentiation of certain subtypes of neutrophil granulocytes into more reactive, hypersegmented subtypes that exhibit delayed apoptosis and increased reactive oxygen species production. Co-colonization of *C. jejuni*-infected neutrophils with intestinal epithelial cells resulted in increased activation of NF-κB signaling pathways in CD16/CD62L subtype human neutrophils and potentially enhanced tumorigenesis (Dolislager et al. 2022).

Three days after infection of ferrets with *C. jejuni*, the maximum concentration of mononuclear phagocytes has been determined (Shank et al. 2018). These migrated mononuclear phagocytes, such as monocytes, enhance the immune response to combat the pathogen by releasing proinflammatory cytokines (Hu and Hickey 2005). For example, after interaction of live or dead *C. jejuni* with THP-1 monocytes, induction of IL-1α, IL-1β, IL-6, IL-8, and TNF-α was observed. Infection-induced translocation of proinflammatory NF-κB toward the nucleus and consequent maturation of THP-1 cells into macrophages are also part of the activated inflammatory processes (Jones et al. 2003). Macrophages will continue to phagocytoze cell debris and bacteria. After early phagocytosis of *C. jejuni*, the pathogen is transported to lysosomes where it is rapidly killed. A protective CCV does not develop in this way (Watson and Galán 2008; Callahan et al. 2021).

Dendritic cells assume both anti- and pro-inflammatory roles during *Campylobacter* enteritis. Contact with the bacterium results in maturation of dendritic cells, which is a preparation of the cell for antigen presentation. The release of IL-10 counteracts an inflammatory response, while the release of proinflammatory chemokines and cytokines further drives inflammation. In addition, the dendritic cells phagocytoze and kill the pathogens and present their antigens. Activation of these immune cells by *C. jejuni* functions by the recognition of LOS by PAMP, which also entails proliferation of B cells and polarization of T cells (Callahan et al. 2021; Hu et al. 2006).

Natural killer cells (NK) also bind to the LOS and inhibit the immune response by subsequent signaling cascades. However, they also participate in the T cell response by antigen presentation. Nevertheless, the maximum concentration of NK cells is observed in experiments with IL-10 − / − mutation only after 7 to 11 days in the affected tissue. Whether an equivalent influence of immune cells could be expected in human enteritis, which is self-limiting on average to one week, cannot be said with certainty (Callahan et al. 2021; Malik et al. 2013).

T cells also increase their numbers at the site of inflammation only from the seventh day after infection in the experimental animals. After main differentiation into T helper 1 cells (Th1), secretion of interferon γ and corresponding cytokines occurs. However, elevated levels of T17 helper cells and lineage-derived cytokines are also detected during infection. T helper 2 cells (Th2), inferred from the low measured concentration of TH2 cytokines, play only a minor role in the pathogenesis of *Campylobacter* enteritis. Vδ1 γδ CD8 + T cells were also observed in higher numbers during infection. They are associated with cytotoxicity and autoimmune responses and thus may be partly responsible for the development of GBS and other autoimmune diseases that may result (Callahan et al. 2021). Elevated titers of Vδ1 cells have been detected in some GBS patients; TLR4 recognition of *C. jejuni* LOS has also been hypothesized (Scelsa et al. 2004; Callahan et al. 2021).

Beginning at day 11 post infection, B lymphocytes maximize the circulating antibody count, directed primarily against *C. jejuni* flagellin as an epitope. The antibodies persist for up to one year after infection (Callahan et al. 2021). In this context, autoreactive IgG1 antibodies represent the largest proportion of predominant immunoglobulins and are associated with the development of GBS (Malik et al. 2013).

GBS is a neurological autoimmune disease associated with *Campylobacter* enteritis, among others, in which ascending paralysis occurs in humans and can be fatal. In this context, GBS is often preceded by an infectious disease, primarily due to *Campylobacter*. Systematic reviews calculated from existing studies that 31% of GBS cases might be due to prior infection with *Campylobacter* (Allos 1997; Poropatich et al. 2010). Mechanistically, suspected causes include cross-reactive immune responses against nerve gangliosides. For example, in GBS patients from Bangladesh, anti-ganglioside IgG antibodies were detected that were equally reactive to the lipooligosaccharides of *C. jejuni*. Furthermore, in the same study, the sugar structures on the gangliosides of the patients and those of LOS from the pathogens were characterized as identical by MS analysis, which could explain the cross-reactivity of the antibodies (Islam et al. 2012).

### The end of Campylobacter enteritis

*Campylobacter* enteritis is generally self-limiting and usually resolves after seven to ten days without the need for antibiotic therapy (RKI 2018). Several factors lead to this circumstance.

*C. jejuni* survives for a maximum of three days within the intestinal epithelium, much shorter than in mononuclear phagocytes (Campana and Baffone 2020; Hickey et al. 2005). To persist, the replication rate of the few actual invading pathogens would have to be correspondingly high. However, replication of *C. jejuni* within invaded epithelial cells, which could compensate for or even exceed pathogen death, was not evident in in vitro experiments with various epithelial cell lines to date. In infected Caco-2, INT 407, and T84 cells, the number of intracellular *C. jejuni* steadily decreases and has already decreased several-fold after 24 h (Campana and Baffone 2020; Buelow et al. 2011; Watson and Galán 2008). At the same time, infected host cells go into programmed cell death due to bacterial-induced apoptosis and thus do not provide a consistent biological niche for the bacterium. Of observed infected 28SC monocytes, more than 50% were already no longer viable after 96 h (Hickey et al. 2005). The described immune reactions of the human body and the associated killing of the pathogens by phagocytes or antibodies, among others, also contribute their part to the relatively rapid eradication (Callahan et al. 2021). Via the resulting diarrhea, in addition to the apoptotically released pathogens, parts of the mucus and accordingly the *Campylobacter* contained therein can also be excreted (Akhondi and Simonsen 2022).

The totality of these parallel processes may explain the self-limitation of *Campylobacter* enteritis over the period of one week on average. Whether the invasion of *C. jejuni* into the intestinal epithelial cell and the resulting apoptotic and immunological processes create an advantage for the pathogen or whether the human as a non-optimal host is to be left again in a fast way would have to be clarified by further investigations.

## Summary and conclusion

*C. jejuni* is an unusual bacterium in many of the aspects considered here: numerous peculiarities of the pathogen have been observed in the entire process of infection over the last 50 years approximately. Infection can be divided into four basic, independent steps that significantly influence virulence.

High, chemotactically controlled motility in the viscous milieu allows targeted and efficient navigation to and within the intestinal mucus with subsequent colonization. By phase variation, *quorum sensing* and extensive posttranslational *O*-and *N*-glycosylation, *C. jejuni* is able to rapidly adapt to environmental conditions. In addition to its classical function as a propulsor, the flagellum is used as a T3SS and becomes relevant again as infection progresses.

Arriving at the intestinal epithelial cell layer, *C. jejuni* utilizes various proteases such as HtrA to first reversibly open cell–cell junctions and subsequently transmigrate paracellularly. Membrane-bound Fn is located at the basolateral side of polarized epithelial cells, which has been one of the most studied cellular binding sites of *C. jejuni* to date. Using the adhesins CadF and FlpA, the pathogen binds to Fn and thus induces intracellular signaling cascades on the one hand, which trigger membrane ruffling and reduced cell migration, among others, via focal adhesions. On the other hand, bacterial-cell contacts of *C. jejuni* secretes invasion antigens by the T3SS, which induce membrane ruffling via a paxillin-independent pathway. Membrane ruffling itself is a typical entry point for pathogens into the host cell because of cytoskeletal restructuring by subsequent engulfment of the bacterium. However, in addition to Fn-binding proteins, other adhesins with other target structures and lectins and their corresponding sugar structures are also involved in adhesion to the host cell.

The uptake or invasion of *C. jejuni* into the intestinal epithelial cell is thereby dependent on certain host cell structures. Fn, clathrin, and dynein thus influence cytoskeletal restructuring, endocytosis, and vesicular transport, respectively, through different mechanisms. A requirement of microtubule and actin structures for efficient invasion is thought to be dependent on the particular cell type invaded. Essential, on the other hand, is their role in the development of membrane ruffling.

In the cell, *C. jejuni* can persist over a 72-h period. Within CCV, the pathogen can escape digestion by lysosomes and enters the perinuclear space via dynein. Meanwhile, various signaling pathways are induced. First, bacterial secretion of CDT directs the cell into programmed cell death. Pyroptotic release of proinflammatory substances then occurs from the now destroyed cell compartments. On the other hand, the immune system reacts to the resulting injuries and the colonized pathogens: an inflammatory cascade with the participation of numerous immune cells of the innate and the acquired immune system develops. Rare development of autoantibodies, possibly directed against both the LOS of *C. jejuni* but also against endogenous gangliosides, may trigger autoimmune diseases such as GBS.

Lesions of the epithelium result in the leakage of electrolytes, water and, in some cases, blood. As a result, symptomatic diarrhea follows, which flushes out mucus containing *C. jejuni* and also the free pathogens. Together with the response of the immune system, this limits the infection to 7 to 10 days in most cases and can be treated with antibiotics only in rare cases.

Based on the enumerated structural interactions between host cell and bacterium, the numerous virulence mechanisms and the resulting signaling cascades and effects that characterize the infection process of *C. jejuni*, a wide variety of targets for attenuation of the pathogen can be characterized.
